# Infection with purified *Piscine orthoreovirus* demonstrates a causal relationship with heart and skeletal muscle inflammation in Atlantic salmon

**DOI:** 10.1371/journal.pone.0183781

**Published:** 2017-08-25

**Authors:** Øystein Wessel, Stine Braaen, Marta Alarcon, Hanne Haatveit, Norbert Roos, Turhan Markussen, Torstein Tengs, Maria K. Dahle, Espen Rimstad

**Affiliations:** 1 Faculty of Veterinary Medicine, Norwegian University of Life Sciences, Oslo, Norway; 2 Norwegian Veterinary Institute, Harstad, Norway; 3 Department of Biosciences, University of Oslo, Oslo, Norway; 4 Faculty of Chemistry, Biotechnology and Food Science, Norwegian University of Life Sciences, Ås, Norway; 5 Norwegian Veterinary Institute, Oslo, Norway; National Cheng Kung University, TAIWAN

## Abstract

Viral diseases pose a significant threat to the productivity in aquaculture. Heart- and skeletal muscle inflammation (HSMI) is an emerging disease in Atlantic salmon (*Salmo salar*) farming. HSMI is associated with *Piscine orthoreovirus* (PRV) infection, but PRV is ubiquitous in farmed Atlantic salmon and thus present also in apparently healthy individuals. This has brought speculations if additional etiological factors are required, and experiments focusing on the causal relationship between PRV and HSMI are highly warranted. A major bottleneck in PRV research has been the lack of cell lines that allow propagation of the virus. To bypass this, we propagated PRV in salmon, bled the fish at the peak of the infection, and purified virus particles from blood cells. Electron microscopy, western blot and high-throughput sequencing all verified the purity of the viral particles. Purified PRV particles were inoculated into naïve Atlantic salmon. The purified virus replicated in inoculated fish, spread to naïve cohabitants, and induced histopathological changes consistent with HSMI. PRV specific staining was demonstrated in the pathological lesions. A dose-dependent response was observed; a high dose of virus gave earlier peak of the viral load and development of histopathological changes compared to a lower dose, but no difference in the severity of the disease. The experiment demonstrated that PRV can be purified from blood cells, and that PRV is the etiological agent of HSMI in Atlantic salmon.

## Introduction

Anthropogenic changes in the rearing of animals, such as large-scale fish farming, may increase the risk of new diseases. Viral diseases pose a significant threat to the productivity in aquaculture [1], and thus to its anticipated share in future global food production. Heart- and skeletal muscle inflammation (HSMI) is an emerging disease in Atlantic salmon (*Salmo salar*) that appeared in the late 1990-ies in Norway, and is characterized by epi-, endo- and myocarditis, myocardial necrosis, myositis and necrosis of the red skeletal muscle [2]. The disease mainly appears a few months after transfer of the fish to seawater. Histopathological lesions in the heart can be found in most fish in an affected sea cage while the cumulative mortality ranges from insignificant to 20% [3].

A viral etiology was suspected and HSMI could be reproduced experimentally by injecting tissue homogenates from diseased fish and transmitted to naïve cohabitants [4]. A novel reovirus, *Piscine orthoreovirus* (PRV), was identified from diseased fish using high-throughput sequencing [5]. PRV is placed in the genus *Orthoreovirus* and has a genome consisting of 10 dsRNA segments distributed in the classical orthoreoviral groups L1–3, M1–3 and S1–4 [6, 7]. Screening has revealed that PRV is ubiquitous in farmed Atlantic salmon in the seawater phase, but high loads of PRV RNA in the heart is a consistent finding during HSMI outbreaks [8]. However, the ubiquitous nature of PRV limits the diagnostic information obtained by RT-qPCR screening. Hence, the association between PRV and HSMI has been questioned.

The main lesions of HSMI are found in the heart, and immunohistochemical detection of PRV in cardiomyocytes demonstrated relationship to HSMI lesions [9]. This supported the hypothesis of PRV being the causative agent of HSMI. Interestingly, erythrocytes were found to be the main target cells for PRV [10]. Piscine erythrocytes are nucleated and contain the transcriptional and translational machineries that enable virus replication. High loads of both PRV RNA and protein can be detected in erythrocytes infected *in vivo* and *ex vivo* [10, 11]. PRV forms viral-like factories in erythrocytes containing viral protein, dsRNA and viral particles and activates the innate antiviral immune response [10–12]. Interestingly, lysis of infected erythrocytes has not been observed *ex vivo* and anemia is not commonly associated with HSMI [11].

So far, HSMI has been reported in farmed Atlantic salmon in Norway, Scotland and Chile [3, 13, 14]. Recently, an outbreak was reported in farmed Atlantic salmon in Canada where HSMI has not previously been reported [15]. However, a number of studies have documented PRV infection in several salmonid species over a wide geographic area without disease association. This includes Pacific salmon (*Oncorhynchus spp*) on the west coast of North-America [16], and wild salmonids (*Salmo spp*) in Norway [17]. Furthermore, an experimental transmission study using PRV-containing material from North-America showed that PRV was transmissible to both Atlantic salmon and Sockeye salmon (*Oncorhynchus nerka*), but neither developed HSMI [18]. PRV has also been detected in archival material dating back to the 1970-ies, before the era of Atlantic salmon farming [16].

Viruses closely related to PRV from farmed Atlantic salmon have recently been discovered in association with diseases in other salmonid species. A virus called PRV-2 was demonstrated to be the possible causative agent of erythrocytic inclusion body syndrome (EIBS), a disease that can cause mass mortality in Coho salmon (*Oncorhynchus kisutch*) [19]. Another PRV-like virus was detected in association with a disease outbreak in rainbow trout (*Oncorhynchus mykiss*) in Norway; the fish displayed signs of circulatory disturbance and histopathological changes resembling HSMI [20]. Furthermore, a PRV strain closely related to the PRV from rainbow trout was found in association with HSMI-like lesions in Coho salmon in Chile [13]. The presence of several PRV variants associated with diseases in salmonids suggests that species adaptation is important for pathogenesis.

The detection of PRV in apparently healthy individuals have spurred speculations regarding the association concerning PRV and HSMI. This includes whether hitherto unidentified co-infecting viruses may contribute to HSMI development [21]. It has also been put forward that host or environmental factors are required for disease development [18]. Altogether, this calls for confirmatory studies to prove a causal relationship between PRV and HSMI. Koch’s postulates were published over 100 years ago to provide a common framework to establish causal relationship between pathogen and disease, but the postulates have their limitations. For instance, the first criteria requires that the organism should only be present in diseased individuals. Based on this alone, the original postulates will never be fulfilled for a large number of diseases in various animal species, including PRV and HSMI. In more recent times, the postulates have been revised to accommodate the current understanding of the host-pathogen relationship [22]. The key experiment to demonstrate causal relationship is to reproduce the disease when a pure culture of the agent is introduced into a healthy host. Virus purification is usually performed following viral propagation in cell culture. However, there are no cell cultures available for the propagation of PRV.

To bypass the lacking possibility to multiply PRV by cell culture propagation, we purified PRV particles directly from infected salmon blood cells, and used the pure virus as inoculum in an experimental challenge trial to prove the causal relationship to HSMI.

## Results

### Propagation of PRV in salmon

**Fig 1:**
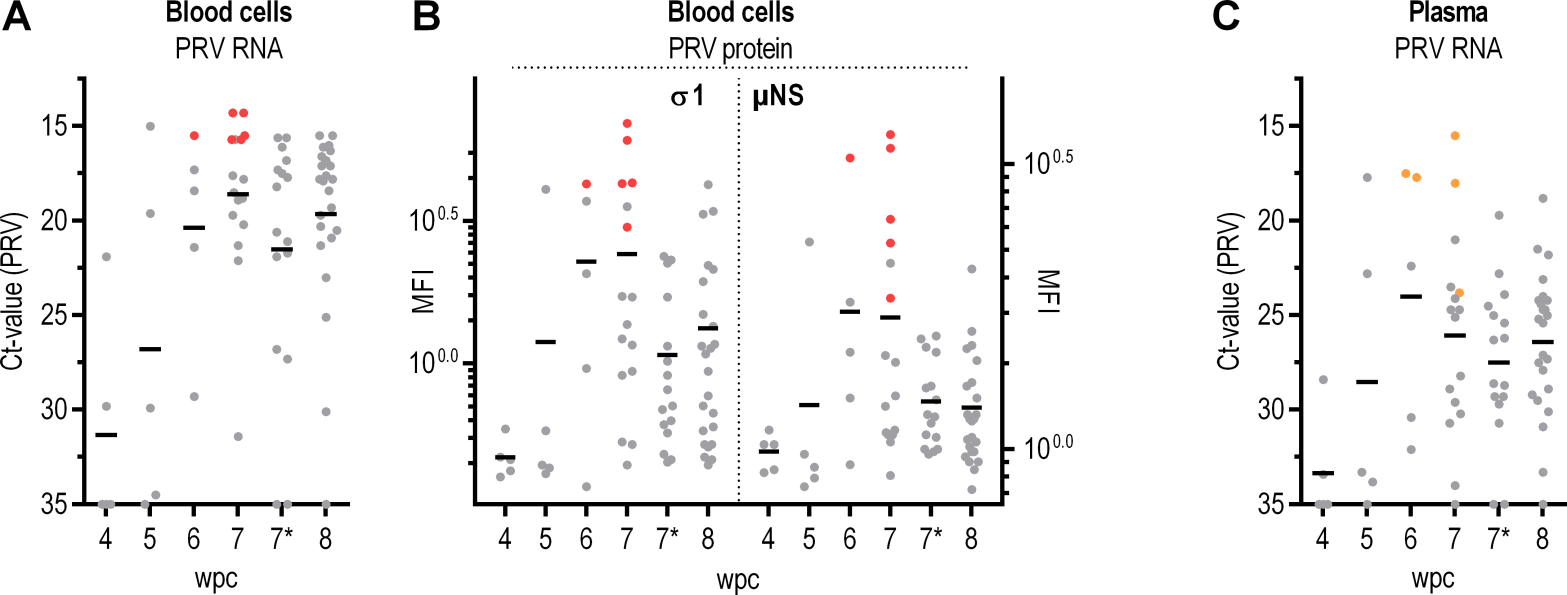
*In vivo* propagation of PRV and selection of samples with high viral load. Viral loads in blood cells and plasma from 4 to 8 weeks post PRV challenge (wpc) in a cohabitation challenge experiment. (A) Blood cells: PRV RNA measured by RT-qPCR, shown as individual and mean Ct-values. (B) Blood cells: Amount of σ1- (left Y axis) and μNS-protein (right Y-axis) measured by flow cytometry, shown as mean fluorescence intensity (MFI) for individual fish and group mean. (C) Plasma: PRV RNA measured by RT-qPCR, shown as individual and mean Ct-values. Color coding: samples selected for PRV purification colored red (A, B: blood cells) or orange (C: plasma). At 7 wpc, two samplings were performed, referred to as 7 and 7* respectively.

Erythrocytes are major target cells for PRV and contain high viral loads in infected Atlantic salmon. We hypothesized that PRV could be purified from blood of infected fish. A cohabitation challenge trial was run where the cohabitant fish were continuously monitored to pin point the time of maximum PRV load in blood (Fig 1). In blood cells, the amount of both PRV RNA and proteins (σ1 and μNS) peaked at 6–7 wpc. From then on, PRV RNA remained high while PRV protein load appeared to drop (Fig 1A, Fig 1B). In plasma, PRV RNA also peaked at 6–7 wpc, but dropped moderately thereafter (Fig 1C). Blood cell- and plasma samples with the highest viral load collected at 6 and 7 wpc were selected for PRV purification (Fig 1).

### Purification of PRV from blood cells and plasma

**Fig 2:**
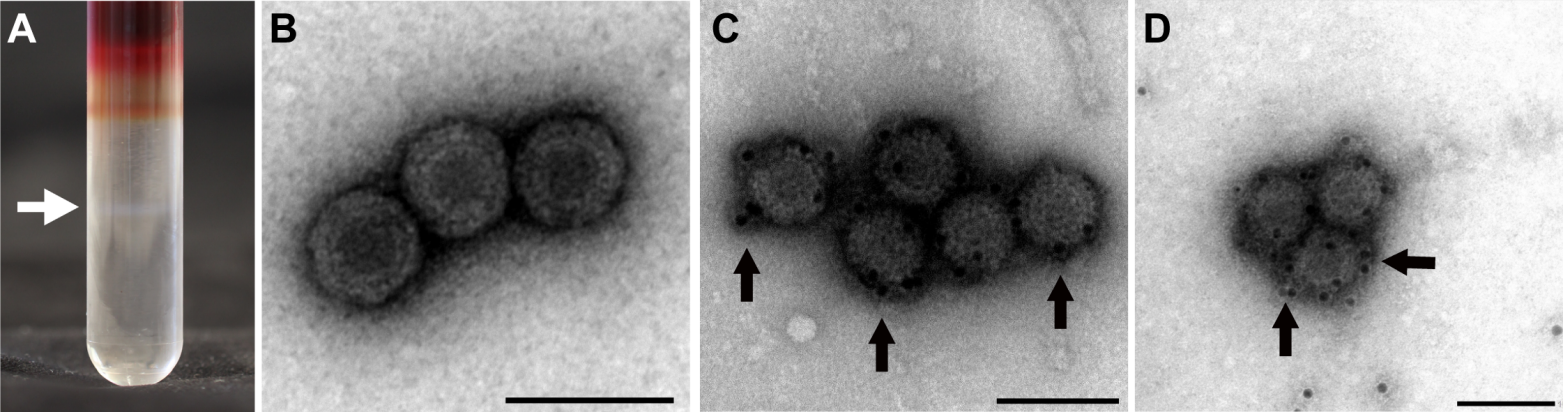
Gradient purification, electron micrographs and immunogold labeling of PRV. (A) Virus band (arrow) detected after CsCl gradient centrifugation. (B) Reovirus-like particles detected by transmission electron microscopy with outer diameter of about 70 nm and inner electron dense core of 39 nm diameter. Scale bar 100 nm. (C) Immunogold labeling of PRV σ1-protein and (D) σ3-protein, both detected in the outer capsid. Scale bar 100 nm.

PRV was purified from the blood cell and plasma samples and viral particles separated on a CsCl gradient. One band at a density of 1.34 (+/-0.01) g/mL was obtained from both blood cells and plasma samples (Fig 2A). The band contained reovirus-like particles as observed by transmission electron microscopy. These were non-enveloped virions with an outer diameter of about 70 nm and an electron dense core with a diameter of 36 nm (Fig 2B). Immunogold labelling demonstrated the presence of both σ1 and σ3 in the putative outer capsid (Fig 2C and 2D).

**Fig 3:**
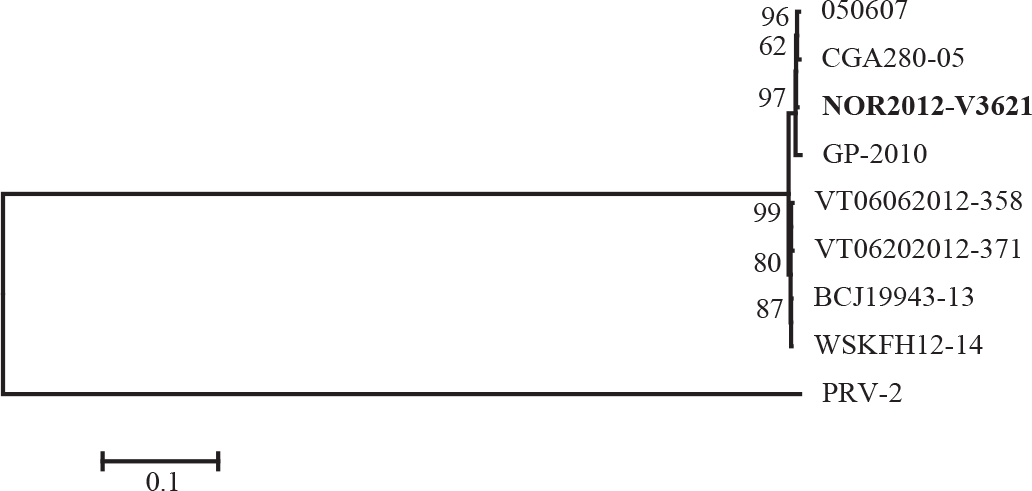
Phylogenetic analysis. Phylogenetic analysis of concatenated coding sequences from nine PRV genomes available in GenBank. The tree was constructed using maximum likelihood and the T92+G model of nucleotide substitution. Bootstrap support for all branches are indicated. PRV-2 was chosen as outgroup. Strain from present study in bold.

High-throughput sequencing of purified PRV particles generated over 43 million reads, with a massive coverage of the PRV genome (> 70 000×). Following assembly, the coding sequences were concatenated and compared with available sequences in GenBank revealing high pairwise sequence identities, both at the nucleotide (98.5–99.9%) and amino acid (99.2–99.9%) levels (S1 Table). Phylogenetic analysis also illustrated the low level of genomic variation, where the present strain grouped together with Norwegian and Chilean strains from HSMI outbreaks (Fig 3).

**Fig 4:**
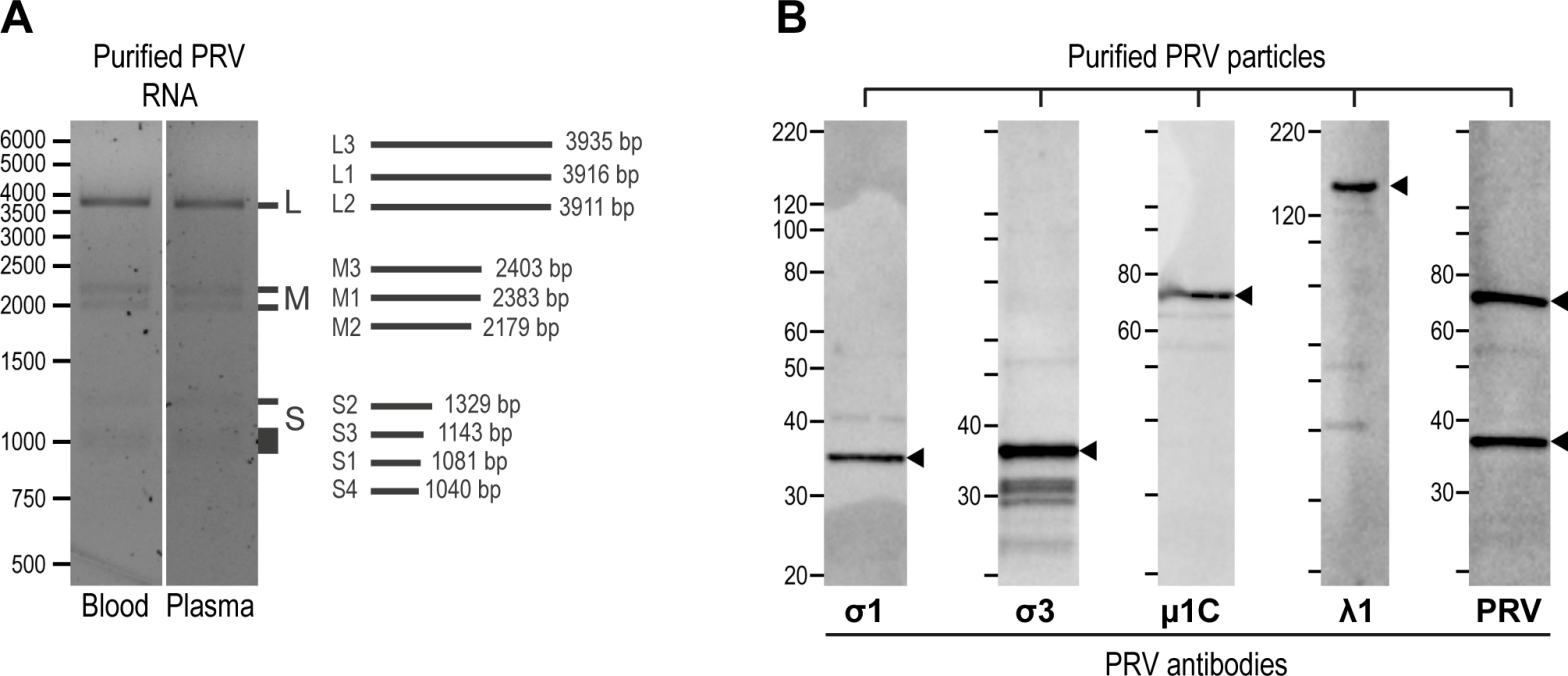
PRV genome and major structural proteins. (A) PRV genomic segments visualized by electrophoresis of RNA extracted from purified PRV, obtained from pelleted blood cells (blood) and plasma, resulting in five separate bands in three molecular weight classes, suggesting co-migration of L1-L3, M1/M3, M2, S2 and S1/S3/S4. Length of each segment as determined by high throughput sequencing is shown on the right. Ladder from 500–6000 bp on the left. (B) PRV particles purified from plasma analyzed by western blotting using protein specific antibodies detecting σ1, σ3, μ1 and λ1 at their predicted full-length size (arrowheads) of 34.6 kDa, 37.0 kDa, 74.2 kDa and 141.5 kDa, respectively. A rabbit antiserum raised against purified PRV particles (PRV) detected the putative full-length μ1 protein in addition to one or both of the σ1- σ3-proteins.

The viral genomic segments were visualized by electrophoresis of RNA extracted from purified PRV. This resulted in five separate bands in three molecular weight classes, suggesting co-migration of L1/L2/L3, M1/M3, M2, S2 and S1/S3/S4 (Fig 4A). The migration pattern is consistent with the length of the individual genomic segments as determined by high-throughput sequencing, assuming that approximately similar sized segments will co-migrate.

Purified PRV particles were analyzed by western blotting using protein specific antibodies, and σ1, σ3, μ1 and λ1 were detected at their predicted full-length size of 34.6 kDa, 37.0 kDa, 74.2 kDa and 141.5 kDa, respectively (Fig 4B). Additional bands were observed, particularly for the σ3 protein, but it is unknown whether these represent degradation products or structural parts of the virus particle. The polyclonal rabbit antiserum raised against purified PRV particles detected the assumed full-length μ1 protein in addition to one or both of the σ1- σ3-proteins (Fig 4B). This serum did not detect the smaller molecular sizes of the σ3-protein.

### Injection challenge with purified PRV; viral load

Two groups of Atlantic salmon were injected with either 2.8×10^8^ particles /fish (PRV-High) or 2.3×10^6^ particles / fish (PRV-Low). Naïve fish were included in each group as cohabitants to confirm transmission of virus.

**Fig 5:**
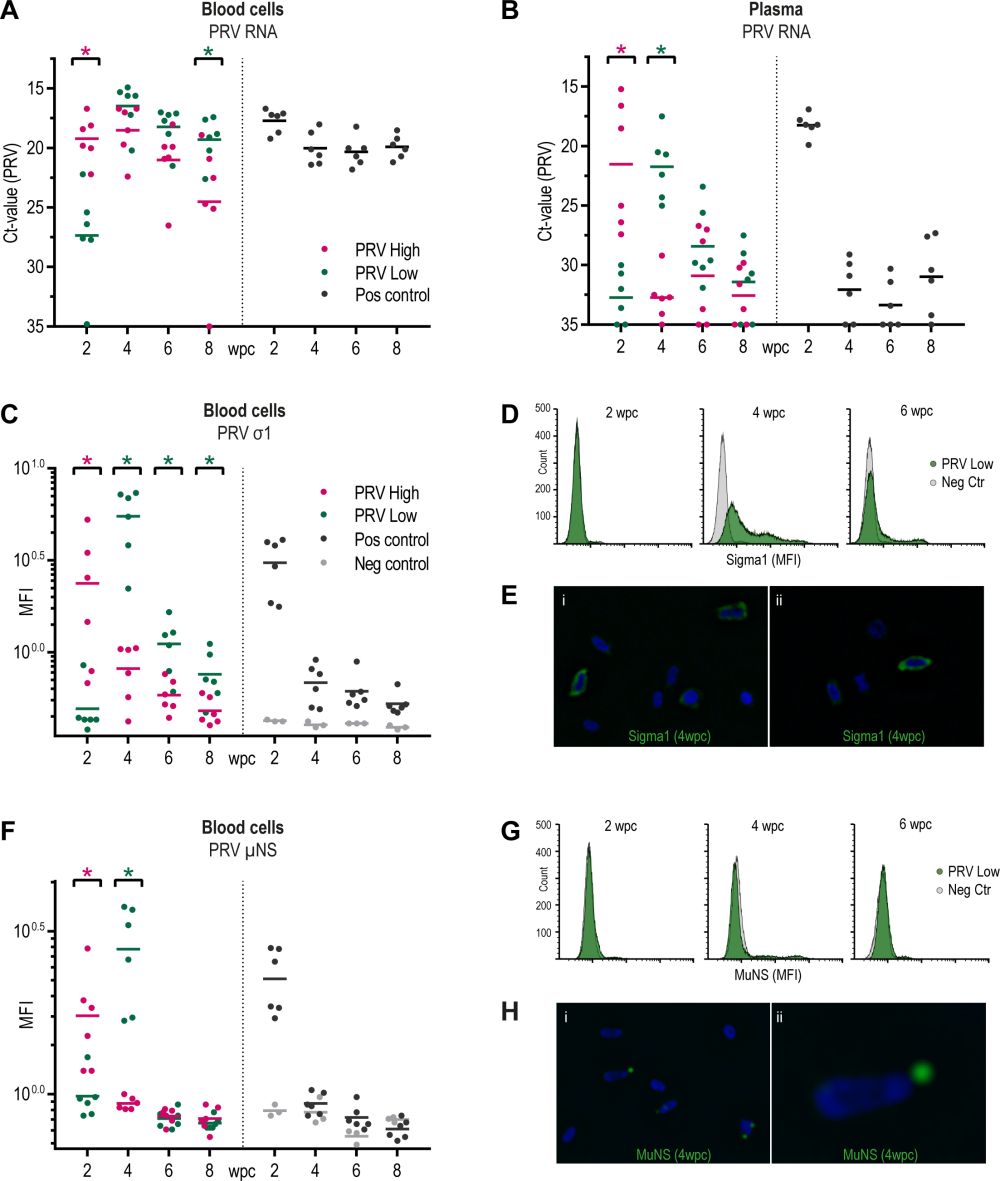
Analysis of blood from injected fish in challenge experiment #2. (A) Blood cells, (B) Plasma: PRV RNA measured by RT-qPCR, shown as individual and mean Ct-values from 2 to 8 weeks post challenge (wpc) (C) Amount of σ1-protein in blood cells measured by flow cytometry, shown as mean fluorescence intensity (MFI) for individual fish and group mean. (D) The results from one individual at 2, 4 and 6wpc from PRV-Low (green) and negative control (grey) groups are presented to illustrate the staining pattern. The fluorescence intensity on X-axis and cell count on y-axis, counting 30 000 cells per sample. (E) Fluorescent labeling of the PRV ơ1-protein (green) in blood cells at 4wpc from PRV-Low. Nuclei stained with Hoechst (blue). The results from μNS staining are presented as for σ1; (F, G) Flow cytometry, (H) Immunofluoresence staining observed by IF microscopy. Statistical analysis comparing PRV-High and PRV-Low was performed using Mann-Whitney test at each time point, *p < 0.05, asterisk color (red and green) indicate the significantly higher group (n = 6).

At 2 wpc, viral RNA load in blood cells was significantly higher for the PRV-High (mean Ct 19.2 ±1.9) compared to the PRV-Low group (mean Ct 27.4 ±4.2) (p < 0.05) (Fig 5A). At 4 wpc, the two groups converged reaching peak viral RNA load; PRV-High (mean Ct 18.5 ±2.3), PRV-Low (mean Ct 16.5 ±2.0). Thereafter, the amount of viral RNA gradually decreased in both groups, although most rapidly in PRV-High. At 8 wpc the viral load was significantly lower in PRV-High (mean Ct 24.5 ±5.6) compared to PRV-Low (mean Ct 19.3 ±1.9) (p < 0.05). In plasma, a significant peak of viral RNA load was observed at 2 wpc in PRV-High (mean Ct 21.5 ±5.4) and at 4 wpc in PRV-Low (mean Ct 21.7 ±2.8), thus reaching similar levels at different time points (Fig 5B). The load of viral RNA in plasma dropped more rapidly than in blood cells. One freeze-thaw process did not appear to reduce the infectivity of the virus (S1 Fig). The positive control group followed a similar pattern as observed in the PRV-High group, but in blood cells the viral RNA level did not drop at the end of the study (Fig 5A and 5B).

Secondly, the amounts of σ1- and μNS-proteins in blood cells of injected fish were analyzed by flow cytometry and immune fluorescence microscopy. (Fig 5C–5H). A significant peak of PRV σ1 protein was observed at 2 wpc and 4 wpc, in PRV-High and PRV-Low respectively, followed by a rapid reduction (Fig 5C). At peak of infection, σ1 appeared to be present in almost all blood cells including a subpopulation containing high amounts of this virus protein (Fig 5D). At later time points, σ1 was detected only at low levels in a minor portion of cells. Positive cells displayed a cytoplasmic staining often as larger globular structures (Fig 5E). The expression of μNS protein coincided with that of σ1, characterized by a significant peak followed by a sharp decline (Fig 5F). At peak of infection a subset of blood cells were μNS positive, but thereafter the protein was undetectable (Fig 5G). The μNS protein was condensed into one or two large globular cytoplasmic inclusions (Fig 5H). The positive control group followed the same development as observed in the PRV-High group, both for detection of σ1 and μNS. Overall, the PRV protein analyses of injected fish indicated the presence of a distinct acute viremic phase, coinciding with high amounts of viral RNA in plasma. Interestingly, the transient nature of this phase was not revealed by RT-qPCR analyses of viral RNA in blood cells.

**Fig 6:**
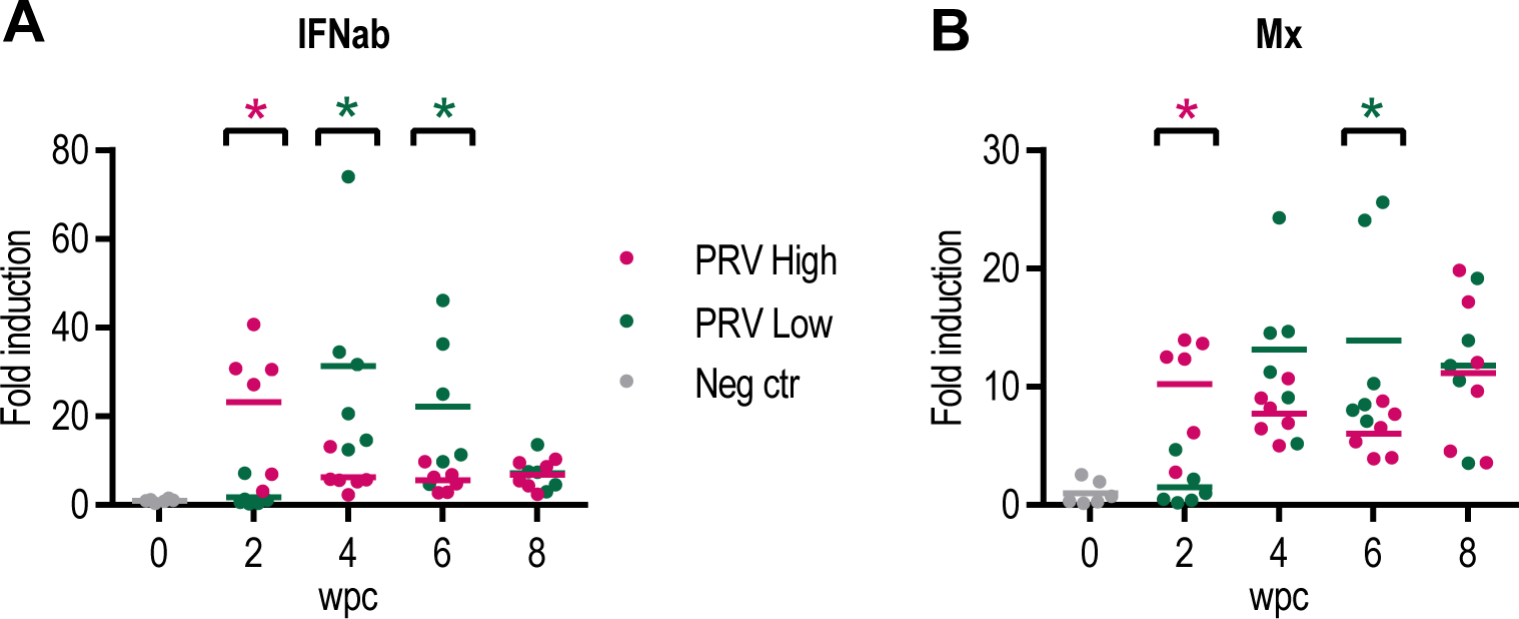
Expression of innate antiviral genes in blood cells in challenge experiment #2. Relative expression of type I interferon (IFNab) (A) and Mx (B) in controls (grey), the PRV-High (red) and the PRV-Low (green) group (n = 6). Target Ct values are normalized against the expression level of elongation factor (EF)1α, and fold induction relative to the mean level of control fish (0 wpc) is shown. Statistical analysis comparing PRV-High and PRV-Low was performed using Mann-Whitney test at each time point, *p < 0.05, asterisk color (red and green) indicate the significantly higher group.

The IFNab and Mx transcription in blood cells of injected fish in PRV-High and PRV-Low were most strongly upregulated at 2 wpc and at 4 wpc, respectively, coinciding with the viremic phase of the infection (Fig 6A and 6B). Thereafter, the transcription of IFNab was reduced, while Mx stayed upregulated throughout the 8 week study period.

**Fig 7:**
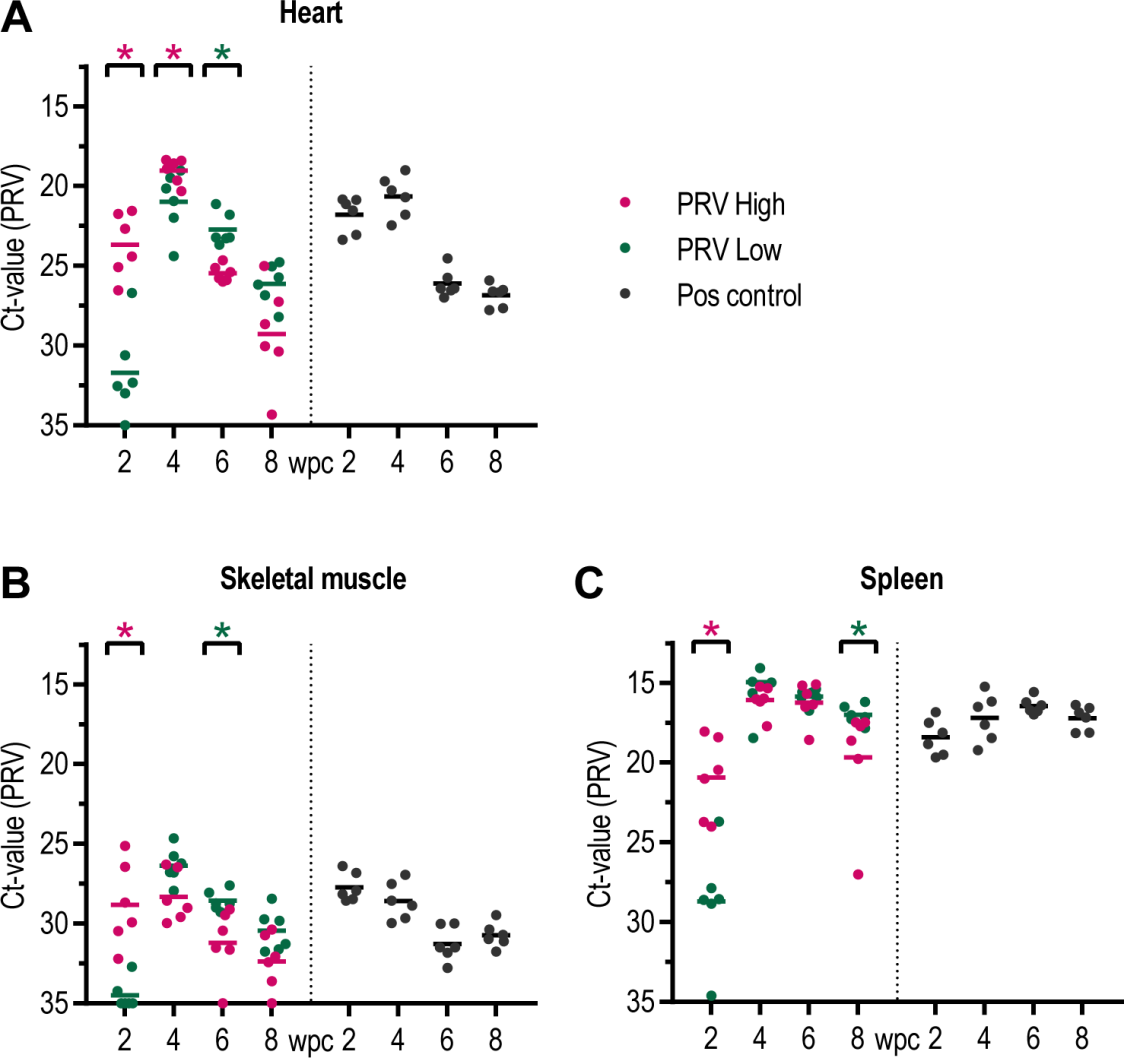
Analysis of PRV RNA in fish injected in challenge experiment #2. (A) Heart, (B) Skeletal muscle, (C) Spleen. PRV RNA measured by RT-qPCR, shown as individual Ct-values and mean from 2 to 8 week post challenge (wpc). PRV-High shown in red, PRV-Low in green and the positive control group in black (n = 6). Statistical analysis comparing PRV-High and PRV-Low was performed using Mann-Whitney test at each time point, *p < 0.05, asterisk color (red and green) indicate the significantly higher group.

The load of viral RNA in the heart, skeletal muscle and spleen of the injected fish showed a similar pattern to that observed in blood cells (Fig 7). In the heart at 2 wpc, the viral load was significantly higher in PRV-High (mean Ct 23.7 ±2.0) compared to PRV-Low (mean Ct 31.7 ±2.8) (p < 0.05), but at 4 wpc both groups reached peak levels with converging viral RNA load, mean Ct 19.0 ±0.8 and 21.0 ±2.0 respectively (Fig 7A). Thereafter, viral RNA levels gradually declined in both groups, but again more rapidly for PRV-High. A similar pattern was observed in the skeletal muscle, with peak infection at 4 wpc but with lower amounts of viral RNA (mean Ct 28.3 ±1.6 and 26.4 ±1.1, respectively) (Fig 7B). In the spleen, rapid increase in PRV-High was observed at 2 wpc, and a coinciding peak in the two groups at 4 wpc with high viral load (mean Ct 16.1 ±0.9 and 15.0 ±2.2, respectively) (Fig 7C). However, in the spleen the viral RNA load remained high. The positive control group followed a similar profile as observed in PRV-High.

### Injection challenge with purified PRV; development of HSMI

**Fig 8:**
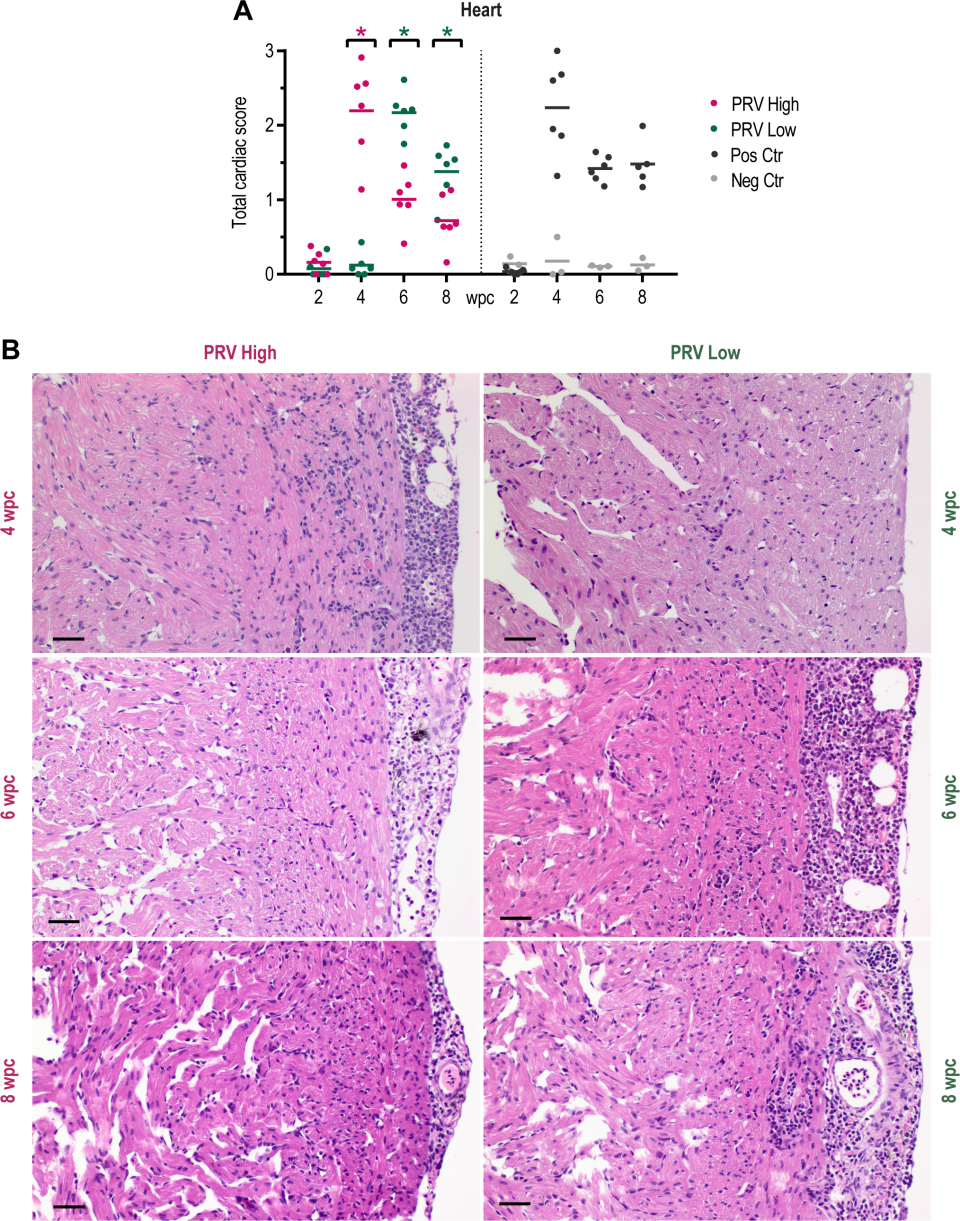
Histopathological lesions consistent with HSMI detected in the heart. Histopathology in the heart of fish injected with purified PRV in challenge experiment #2. (A) Histopathological score shown as individual score (total cardiac score) and group mean from 2 to 8 weeks post challenge (wpc) for the PRV-High (red), PRV-Low (green), positive control (black) and negative control (grey) group (n = 6). Statistical analysis comparing PRV-High and PRV-Low was performed using Mann-Whitney test at each time point, *p < 0.05, asterisk color (red and green) indicate the significantly higher group. (B) Light microscopic images of cardiac histopathology in representative fish at 4, 6 and 8 wpc from PRV-High (red) and PRV-Low (green) to illustrate development of HSMI. Ventricle showing epicarditis and myocardial inflammation in the compact myocardium. Scale bar 20 μm.

The histopathological lesions in the heart first appeared and peaked at 4 wpc in PRV-High and at 6 wpc in PRV-Low with mean total cardiac score of 2.2 ±0.6 and 2.2 ±0.3, respectively (Fig 8A). The lesions observed were consistent with those of HSMI (Fig 8B). In subsequent samplings, there was a reduced total cardiac histopathologic score. The scoring of each of the separate compartments (epicardium, compactum, spongiosum, atrium) reflected that of the total cardiac score (S2 Fig). In the positive control group, the lesions followed a similar pattern as PRV-High, but did not subside as rapidly.

**Fig 9:**
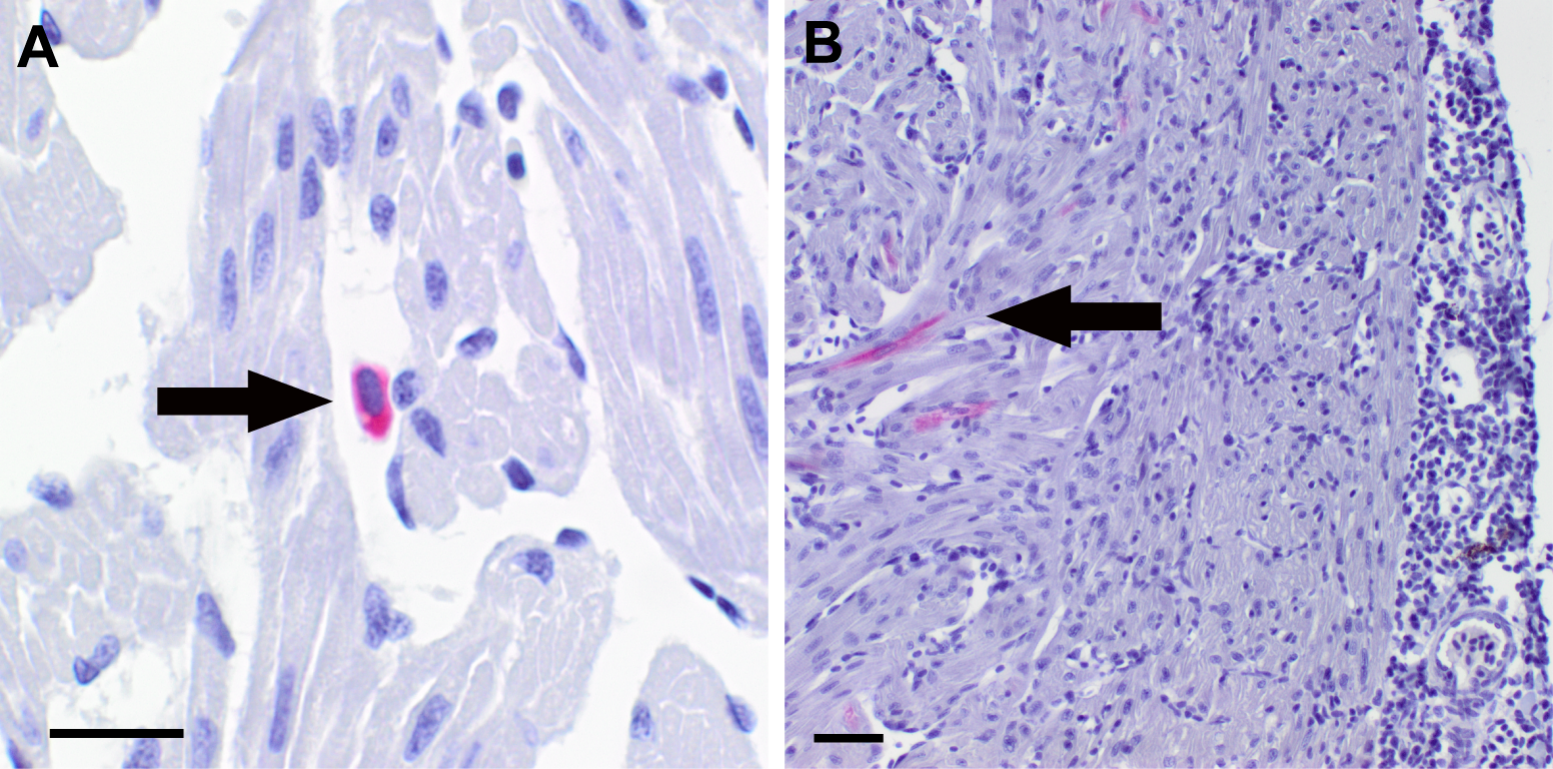
Immunohistochemical detection of PRV in heart sections of infected fish. Immunohistochemical staining of PRV in heart sections from the PRV-High group in challenge experiment #2, using σ1-antibodies. (A) Positive erythrocyte (arrow) detected at 2 wpc as even cytoplasmic staining (red). Scale bar 10 μm. (B) PRV antigen (red) detected in cardiomyotes (arrow) in the heart ventricle at 4 wpc. Scale bar 20 μm.

Immunohistochemistry of heart sections from PRV-High demonstrated the presence of PRV antigen. At 2 wpc, positive erythrocytes were observed in the heart of one out of six fish (Fig 9A), coinciding with peak amount of viral protein observed by flow cytometry. At 4 wpc, PRV antigen was detected in cardiomyocytes in four out of six fish (Fig 9B), coinciding with the maximum of histopathological lesions and PRV RNA in the heart of this group. PRV antigens were detected as evenly distributed cytoplasmic staining in erythrocytes and cardiomyocytes in both the compactum and the spongiosum layers. PRV antigen was not detected in the subsequent samplings (6 wpc, 8 wpc) where the histopathological lesions were subsiding and the loads of PRV RNA were reduced.

**Fig 10:**
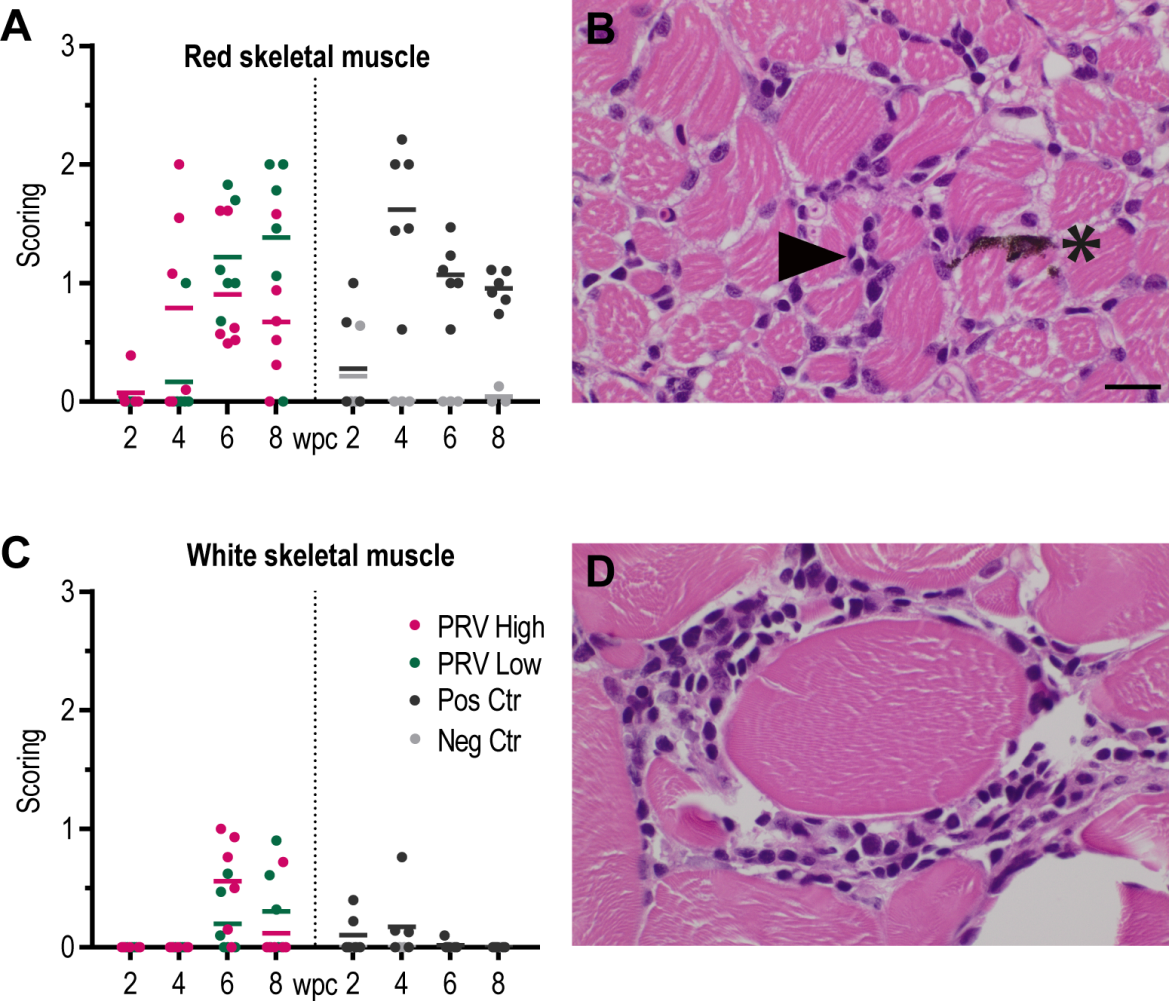
Histopathological lesions consistent with HSMI detected in skeletal muscle. Histopathology in the skeletal muscle of fish injected with purified PRV in challenge experiment #2. (A) Histopathological score in red skeletal muscle shown as individual and mean score from 2 to 8 weeks post challenge (wpc) for the PRV-High (red), PRV-Low (green), positive control (black) and negative control (grey) group (n = 6). Statistical analysis comparing PRV-High and PRV-Low was performed using Mann-Whitney test at each time point, p < 0.05. No significant difference observed. (B) Light microscopic image of red skeletal muscle, 6 wpc, PRV-Low group. Mild myositis (arrowhead) and presence of melanin (*). Scale bar 10 μm. (C) Histopathological score in white skeletal muscle shown as individual and mean score, presented as for red skeletal muscle. (D) Cross section of white skeletal muscle, 6 wpc, PRV–High; Mild inflammation around white muscle fiber.

The first histopathological lesions in red skeletal muscle appeared to coincide in time with that of heart; at 4 wpc in PRV-High and at 6 wpc in PRV-Low (Fig 10A). At 6 wpc lesions were detected in all fish in both groups, with a mean score of 0.9 ±0.5 in PRV-High and 1.2 ±0.4 in PRV-Low. At 8 wpc the severity of the lesions in PRV-High appeared to subside, while in PRV-Low they were increasing. In general, the lesions were not as severe as those in the heart and were characterized by mild to moderate inflammation between fibers and necrotic myocytes (Fig 10B). Mild amount of melanin was observed in red muscle of some fish. Lesions in the red skeletal muscle of the positive control group peaked at 4wpc, then subsided to 8wpc.

Few and minor lesions were also detected in white muscle of some individuals in both groups at 6 wpc and 8 wpc (Fig 10C). These were characterized by degeneration of the myocytes and cellular infiltration between the fibers. Little or no lesions were detected in the white skeletal muscle of the positive control group (Fig 10D).

### PRV is transmitted to naïve fish which develop HSMI

**Fig 11:**
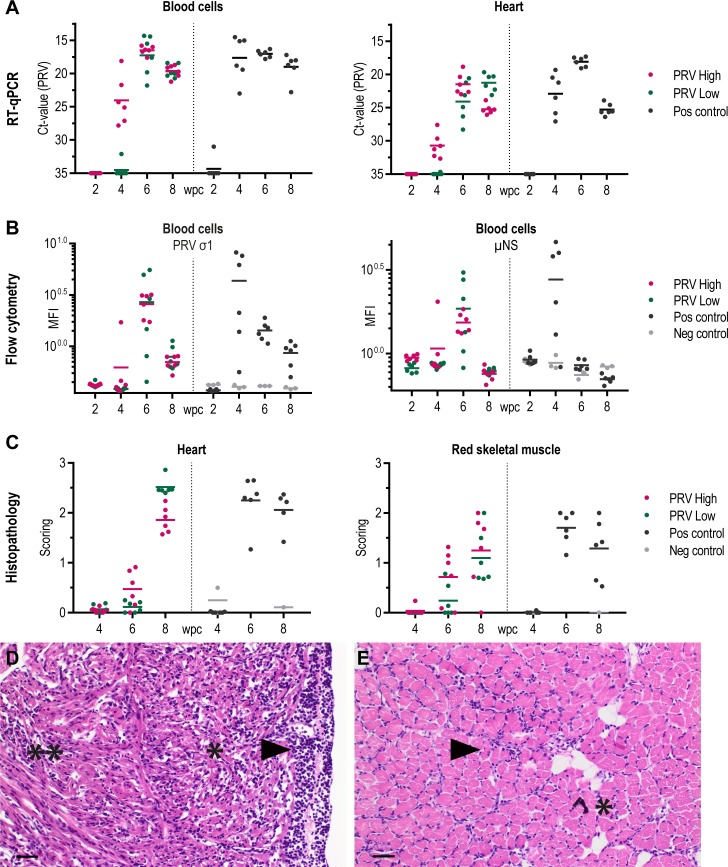
Summary cohabitant group. (A) PRV RNA in blood cells and heart measured by RT-qPCR, shown as individual and mean Ct-values from 2 to 8 weeks post challenge (wpc). (B) Amount of σ1- and μNS -protein measured by flow cytometry, shown as mean fluorescence intensity (MFI) for individual fish and group mean. (C) Histopathological score in the heart (total cardiac score) and red skeletal muscle shown as individual and group mean from 4 to 8 wpc. (D) Light microscopic images at 8 wpc in PRV-Low; (D) Heart lesions in the ventrical including severe epicarditis (arrowhead) and inflammation in the compact (*) and spongy myocardium (**) consistent with HSMI. (E) Mild inflammation in the red skeletal muscle (arrowhead), presence of melanin (*). Scale bar 20 μm. Color coding: PRV-High (red), PRV-Low (green), positive control (black) and negative control (grey). n = 6.

In naïve fish cohabitating with the injected fish, PRV RNA and protein were detected in blood and heart, demonstrating transmission of virus (Fig 11A and 11B). The viral kinetics in naïve fish displayed a similar pattern to that of injected fish, but the delay in time of onset between PRV-High and PRV-Low was reduced.

At 4 wpc, PRV RNA was detected in blood cells and heart of all cohabitant fish in PRV-High, but in only one cohabitant fish to the PRV-Low group (Fig 11A). At 6 wpc, the viral RNA load in blood cells of the two groups converged, and this coincided with peak load of viral proteins (Fig 11B). A similar pattern was also observed in the heart. At 8 wpc, the viral load in blood cells appeared to decrease in both groups, but this was more pronounced for PRV protein than RNA. In the heart at 8 wpc, viral RNA levels decreased in PRV-High cohabitants, but continued to increase in PRV-Low. The positive control group in general followed a similar pattern as PRV-High. Histopathological lesions consistent with HSMI were observed in cohabitants of both PRV-High and PRV-Low at 8 wpc (Fig 11C–11E). PRV was successfully purified from blood cell samples collected during peak of infection from both injected and cohabitant fish (data not shown).

## Discussion

This report shows that purified PRV particles induce heart and skeletal muscle inflammation (HSMI) in Atlantic salmon. Electron microscopy, western blot and high-throughput sequencing all verified the purity of the viral particles. The purified virus replicated in infected salmon and histopathological changes consistent with HSMI were observed in both injected and cohabitant fish. PRV specific staining in the pathological lesions was demonstrated. A dose dependent response was evident as the lower dose of virus gave a delay in both the peak viral load and development of histopathological changes. However, neither the maximum viral load nor severity of the lesions differed between the PRV-High and PRV-Low groups. The Koch’s original causality postulates stated that a pathogen should induce the disease after experimental infection of a pure culture and be re-isolated from the experimentally infected animal. These criteria are fulfilled, thus PRV is the etiologic agent of HSMI in Atlantic salmon.

The Koch’s postulates require that the inoculum used in experimental infection should consist of purified virus following propagation in cell culture, which is not possible for PRV. To bypass this requirement, we purified virus from blood cells and plasma of infected fish. The key to this success is that erythrocytes, which are numerous and readily available for collection, are the main target cells for PRV replication. Blood from PRV-infected fish was collected during peak of infection; i.e. at the highest levels of virus proteins and -RNA in blood cells. Following peak of infection, there was a rapid decline in virus proteins in blood cells. Samples collected after this short period did not yield any virus band in the gradients after the purification procedure.

In our study, the virus particles were purified, quantified by RT-qPCR, shown to be infectious, but we could not measure the titer of infectious virus. Nevertheless, purified quantitated viral particles provide a significantly improved model compared to previously used tissue homogenates as inoculum. Furthermore, one freeze thaw cycle did not significantly reduce the infectivity, which is important for storage of the pure virus fraction. We cannot distinguish between inoculated viral RNA and newly synthesized viral RNA. High loads of PRV RNA was detected at 2 wpc in the PRV High group, which coincided with peak loads of the non-structural protein μNS. Non-structural proteins are not a part of purified virus particles but synthesized in infected cells, and thus we conclude that at 2 wpc the PRV replication in the PRV High group was high. Purified virus will enable physical characterization, including inactivation studies, which could be of importance for management and biosecurity in fish farming. Purified PRV will also be vital for cell culture studies. In addition, it offers the ability to test the efficacy of an inactivated whole virus vaccine against HSMI.

The kinetics of PRV RNA and PRV protein differed in blood cells. Up until peak infection, both PRV RNA and protein levels increased in blood cells. However, post peak infection, viral protein levels dropped rapidly while RNA levels remained relatively high. The peak of PRV protein in blood cells appeared in a relatively short window of time coinciding with a distinct increase of PRV RNA in plasma, which indicates release of viral particles. Histopathological lesions were consistently detected after the acute phase. This is consistent with previous results which have indicated that PRV infection of blood cells is an acute infection; at peak of infection a number of PRV proteins can be detected in blood cells accompanied by high loads of PRV RNA, whereas post peak infection the viral protein drops substantially while RNA levels persist at high levels [23]. Quantitative analysis of PRV RNA should be interpreted with caution and preferably be accompanied by PRV protein analysis. Further research is needed to investigate the nature of the putative persistent PRV infection of blood cells.

The peak phase in virus production in blood is accompanied by expression of IFNab and the IFN-regulated gene Mx. Whereas IFN is regulated by viral infection by cell signaling induced via pattern recognition receptors, Mx is primarily regulated secondary to interferon production by another signaling pathway [24]. For this reason the kinetics of IFNab and Mx regulation differ, which has also been shown previously in a transcriptomic study of PRV-infected erythrocytes [12]. The antiviral response may be the cause of the drop in viral protein and virus release into plasma, as several of the IFN-regulated gene products serve to block virus protein synthesis and virus release [24].

Phylogenetic and sequence analyses have earlier indicated that PRV is a fiber-encoding, nonfusogenic orthoreovirus [6, 7]. These are both properties of the assumed last common ancestor of both orthoreoviruses and aquareoviruses estimated to have existed around 510 million years ago [25, 26]. The morphology of the PRV virion i.e. spherical and approximately 70 nm diameter, and three major structural proteins making up the outer virion coat, as indicated by the reactivity of the whole virus antiserum, resemble ortho- and aquareoviruses [27]. However, PRV possesses 10 genomic segments, which is a key feature that differs the orthoreoviruses from the aquareoviruses and the buoyant density in CsCl of PRV was found to be 1.34 (+/-0.01) g/mL, which is less than the 1.36 g/mL of both ortho- and aquareoviruses. The placement of PRV at the root of the *orthoreovirus* and *aquareovirus* genera indicate that further characterization of PRV may reveal information not only for fish virology but also for the evolution and speciation of reoviruses and their hosts.

The association between PRV and HSMI has been questioned, and speculations whether other, unidentified viruses could contribute to disease development has been put forward [18, 21]. PRV is ubiquitous in farmed Atlantic salmon both in Norway and western Canada. However, HSMI outbreaks are prevalent in Norway but have hardly been reported in Canada [8, 15, 16]. Furthermore, HSMI has earlier been reproduced experimentally after challenge with tissue homogenates from fish with HSMI in Norway [4], whereas challenge with tissue homogenates from PRV infected fish in Canada has not induced HSMI [18]. These differences could be due to properties of the virus, host, and/or management. The widespread distribution of HSMI in Norway indicate that all current breeds of Atlantic salmon are susceptible to disease development, and no specific management procedures that could be linked to HSMI have been identified. Suboptimal management, other infections, handling, sorting, treatments and transport are all challenges for industrialized farming of fish and influence the outcome of infectious diseases.

Alignment of full-length genome sequence of the PRV strain used in the present study showed that it grouped with PRV sequences obtained from HSMI outbreaks. It would be interesting to do further experimentation with challenge dosage, to determine whether there exists a threshold that would produce PRV positive, but HSMI negative fish. Analyses of a small number of whole genome [28] and a larger number of partial S1 sequences have so far not identified specific genotypes related to virulence [13, 29, 30]. To single out putative differences between viral strains in their ability to induce HSMI, i.e. differences in virulence, whole genome sequencing and standardized challenge studies, using identical input of purified PRV, are required.

The difference in prevalence of HSMI in farmed Atlantic salmon in British Colombia, Canada, and Norway is enigmatic. In our study we found strong induction of the innate antiviral response as measured by transcription of IFNab and Mx, as also previously observed [12]. Interestingly, in a Canadian experimental infection trial, high levels of PRV RNA was detected in the fish, but only minor effects on the innate antiviral response was obtained and HSMI did not develop [18]. Together, this suggests a role of the early innate antiviral response in the pathogenesis of HSMI and that the capacity to induce this response differs between PRV strains.

The present study confirms that PRV infection causes HSMI in Atlantic salmon, but it does not suggest that all strains of PRV can induce HSMI. The ability to purify PRV enables further characterization of PRV strain differences.

## Material and methods

### Challenge experiments

#### Ethics statement

Two challenge experiments were conducted at VESO Vikan aquatic research facility (Vikan, Norway). Both experiments had been approved by the Norwegian Food Safety Authority (NFDA) according to the European Union Directive 2010/63/EU for animal experiments (permit number 8446 and 8658). The immunization of a rabbit to produce of polyclonal antibodies was performed at the Section for Experimental Biomedicine at The Norwegian University of Life Sciences in Oslo, Norway. The unit is licensed by the Norwegian Animal Research Authority (NARA) and accredited by Association for Assessment and Accreditation of Laboratory Animal Care. The study was approved by the unit’s animal ethics committee (Institutional Animal Care and Use Committee/IACUC) and NARA (permit number 7487) according to the European Union Directive 2010/63/EU and Norways own regulation based on the EU directive The regulation on use of animals in research FOR-2015-06-18-761.

#### Experimental fish

The study included two separate challenge experiments conducted at VESO Vikan aquatic research facility (Vikan, Norway). In both experiments, fish were acclimatized for one week prior to challenge, fed according to standard procedures and kept under 24 h light regime. The experimental fish, Atlantic salmon of a SalmoBreed Optimal strain (Bergen, Norway), were confirmed free of known salmon pathogens before initiation of the study. The fish were anesthetized by bath immersion in benzocaine chloride (2–5 min, 0.5 g / 10 L water) prior to handling, and euthanized using concentrated benzocaine chloride (1 g / 5 L water). The fish were observed minimum once per day. The criterion used to exercise humane endpoint was that affected fish had lost their ability to maintain an upright position in the tanks and were not evasive to netting. Any fish that reached the terminal stage when fish were tendered were euthanized. The fish used in the trial were from a healthy population and no fish died during the course of the trial other that those sampled or died from disease or humane endpoint.

#### Challenge experiment #1: PRV propagation and purification

The experiment included 162 unvaccinated seawater-adapted Atlantic salmon with an initial average weight of 160 grams. During the challenge, the fish were kept in a tank supplied with particle filtered and UV treated brackish water (15 ‰ salinity, 12°C) up to 3 weeks post challenge (wpc). The fish were then switched to seawater (34 ‰ salinity, 12°C) for the remaining time of the study. The inoculum, consisting of pelleted blood cells, originated from a HSMI outbreak in 2012 and had undergone one previous passage in experimental fish. The inoculum has previously been described [31]; it contained high loads of PRV as analyzed by RT-qPCR, and was confirmed negative for infectious pancreatic necrosis virus (IPNV), infectious salmon anemia virus (ISAV), salmonid alphavirus (SAV) and piscine myocarditis virus (PMCV). Prior to inoculation, the blood pellet was diluted 1:1 in PBS.

A cohabitation challenge was run with 75 shedder fish injected intraperitoneally with 0.1 mL inoculum, marked by shortening of the adipose fin and placed in a tank containing 75 naïve fish (cohabitants). To estimate the peak of infection, heparinized blood was taken from the caudal vein of five cohabitant fish weekly, from 3 wpc to 6 wpc. At peak of infection, remaining fish were sampled on three subsequent occasions; 7 wpc (n = 16), 7 wpc + 2 days (n = 16) and 8 wpc (n = 24). At each sampling, two non-infected fish of the same population kept in a separate tank served as negative controls. In addition, blood samples were collected from five fish before initiation of the experiment (0 wpc).

The heparinized blood was immediately analyzed for PRV by flow cytometry, as described below. The blood was then centrifuged at 3000 × g for 5 min at 4°C, separating plasma and blood cells. Both were analyzed for PRV by RT-qPCR and the remaining samples were stored for PRV purification (details described below).

#### Challenge experiment #2: Causality between PRV and HSMI

The experiment included 204 unvaccinated seawater-adapted Atlantic salmon with an initial average weight of 64 grams. During the challenge, the fish were kept in a tank supplied with particle filtered and UV treated seawater (34 ‰ salinity, 12°C)

The experiment included four groups; a PRV-High and PRV-Low group challenged with purified PRV from challenge experiment #1, using a high and low (120-fold lower) dose respectively; a positive-control group challenged with a standard, non-purified PRV homogenate and a negative-control group of naïve fish. The four groups were kept in separate tanks in which 24 fish were challenged by injection, labeled by shortening of the adipose fin, and 24 naïve cohabitant fish were added.

The inoculum of purified PRV was prepared by pooling batches of purified PRV in PBS from challenge experiment #1 and the number of particles was quantified by RT-qPCR using an absolute quantification standard curve (described below). The PRV-High group was injected with 2.8×10^8^ particles per fish; administered as 100 μL intraperitoneal (i.p.) and 50 μL intramuscular (i.m.) injection. The PRV-Low group was injected with 2.3×10^6^ particles per fish; administered as 75 μL i.p. and 50 μL i.m. injection, thus a 120-fold lower dose.

The inoculum for the positive control group was a blood cell pellet collected from challenge experiment #1, diluted 1:5 in L15 medium Leibovitz’s L15 medium (Life Technologies) supplemented with gentamicin at 50 μg/mL (Gibco, Life Technologies, Grand Island, NY, USA) and Fungizone at 0.25 g/mL (Gibco), sonicated five times at 20 kHz for 30 sec on ice and centrifuged at 5000 × g for 10 min at 4°C. The resulting supernatant was shown to contain high viral loads as determined by RT-qPCR (Ct-value 22.0). The absolute quantification assay was not applied for this inoculum as the total RNA contained a mix of viral genomic RNA and transcripts. The positive control group was injected with the blood pellet supernatant administered as 100 μL i.p. and 50 μL i.m. injection. The negative control group was left untreated.

At each sampling, heparinized blood from the caudal vein was collected for PRV analysis by RT-qPCR and flow cytometry, and for PRV purification. In addition, tissue from heart, skeletal muscle and spleen were sampled in RNAlater (Life Technologies, Carlsbad, CA, USA) for RT-qPCR analysis and parallel samples from the heart and skeletal muscle were harvested in 10% phosphate buffered formalin and used for histological analysis.

To evaluate the effect of the freeze/thaw cycle on purified PRV, an additional group called PRV-Low-Frozen was included. The set up was identical to PRV-Low, challenging with the same amount of PRV, in the same volume and by the same route. However, the inoculum was prepared with 15% glycerol and frozen for 24 h at -80°C. From this group, only heparinized blood was collected and analyzed for PRV by RT-qPCR and flow cytometry.

### PRV analysis

#### RNA isolation

Total RNA was isolated from tissue samples (25 mg) and pelleted blood cells (20 μL). The samples were homogenized in QIAzol Lysis Reagent (Qiagen, Hilden, Germany) using 5 mm steel beads and TissueLyser II (Qiagen) for 2 × 5 min at 25 Hz, added chloroform and centrifuged. Collected the aqueous phase and proceeded with automated RNA isolation using RNeasy Mini QIAcube Kit (Qiagen) as described by the manufacturer. The RNA was quantified using a NanoDrop ND-1000 spectrophotometer (Thermo Fisher Scientific, Waltham, MA, USA).

For cell free samples, including plasma and samples of purified virus, a combination of Trizol LS (Life Technologies) and RNeasy Mini spin column (Qiagen) was used. Briefly, 10 μL sample was diluted in 130 μL PBS, mixed with Trizol LS, added chloroform, then separating the phases by centrifugation. The aqueous phase was collected and proceeded with the RNeasy Mini spin column (Qiagen) as recommended by the manufacturer, eluting isolated RNA in 50 μL RNase-free water.

#### Absolute quantification of PRV

The open reading frame (ORF) of the PRV S1 genomic segment encoding the σ3 protein was cloned into the pcDNA3.1 vector (Invitrogen, Eugene, OR, USA), linearized downstream of the σ3 gene with EcoRV, and *in vitro* transcribed at 15°C for 2 h to optimize for long transcripts using the Ambion MAXIscript T7 In Vitro Transcription Kit (Life Technologies) following the manufacturers protocol. Samples were treated with DNase (Life Technologies) and purified on NucAway columns (Ambion, Foster City, CA, USA). Transcript quality was analyzed on a bioanalyzer using RNA nano chip analysis (BioRad, Hercules, CA, USA), the concentration (ng/μL) were measured on a NanoDrop ND-1000 spectrophotometer (Thermo Fisher Scientific), and the number of transcripts per μL calculated using the formula: Concentration transcript (ng/μL): (number of nucleotides × 320,5 + 159 (ng/nmole) = nmole transcripts/μL, Mole transcripts/μL × 6.02214×10^23^ transcripts/mole (Avogadros #) = Number of transcripts/μL. Samples were then diluted to 10^9^ transcripts/μL, added RNaseOut (Life Technologies) and stored in 5μL aliquots at -80°C. Prior to cDNA synthesis, a seven point standard curve was prepared from 10-fold serial dilutions of transcripts, resulting in 10^8^ to 10^2^ transcripts per μL.

#### RT-qPCR for PRV

The Qiagen OneStep kit (Qiagen) was used for RT-qPCR for PRV. The input was standardized to 100 ng (5 μL of 20 ng/uL) of total RNA per reaction for tissues (heart, skeletal muscle, spleen) and blood cells. For cell free samples, i.e. plasma and purified virus samples, 5 μL out of the 50 μL eluted RNA was used as input. Prior to RT-qPCR, the template RNA was denatured at 95°C for 5 min. The RT-qPCR targeted PRV segment S1 and performed using previously described conditions [11]. The samples were run in duplicate, and a sample was defined as positive if both parallel samples had a Ct < 35. Primers and probe are listed in Table 1.

**Table 1:** Primers and probe used in this study.

Primer/probes	Primer sequence (5’ → 3’)	Genbank acc. no.
ơ3 S1-659 Fwd	TGCGTCCTGCGTATGGCACC	GU994022
ơ3 S1-801 Rev	GGCTGGCATGCCCGAATAGCA	
ơ3 S1-693 Probe	*FAM-*ATCACAACGCCTACCT–*MGBNFQ*	
IFNab Fwd	CCTTTCCCTGCTGGACCA	DQ354152
IFNab Rev	TGTCTGTAAAGGGATGTTGGGAAAA	
Mx Fwd	GATGCTGCACCTCAAGTCCTATTA	XM_014133086
Mx Rev	CACCAGGTAGCGGATCACCAT	
EF1α Fwd	TGCCCCTCCAGGATGTCTAC	BG933897
EF1α Rev	CACGGCCCACAGGTACTG	

The quencher and reporter dye of the probes are in italic. Accession numbers are shown in the right column.

#### Flow cytometry

Blood cells were analyzed for PRV proteins σ1 and μNS by flow cytometry with separate staining for each viral protein. All steps were performed on ice. Heparinized blood was diluted 1:20 in staining buffer (PBS + 1% BSA + 0.05% azide), plated into 96-well plates as 50 μL aliquots and washed in staining buffer. The cells were fixed in IC Fixation Buffer (eBioscience, San Diago, CA, USA) and washed in Permeabilization Buffer (eBioscience). The blood cells were stained for 30 min using polyclonal antibodies raised against PRV σ1 (Anti-σ1, #K275) [9] or PRV μNS (Anti-μNS #R320684) [23], using a 1:5000 and 1:2000 dilution respectively. Finally, the cells were stained with secondary Alexa Fluor 488 conjugated anti-rabbit IgG (Molecular Probes, Eugene, Oregon, USA) (2 mg/mL diluted 1:800) for 30 min. The blood cells were read on a Gallios Flow Cytometer (Beckman Coulter, Miami, FL, USA) counting 30 000 cells per sample, and the data were analyzed using the Kaluza software (Becton Dickinson). Corresponding sera collected prior to immunization was used as a negative control to compensate for slight variation in the background staining of each sample.

#### Immunofluorescence microscopy

Blood cells stained with PRV σ1 and μNS antibodies (used in flow cytometry) were also inspected by immunofluorescence microscopy. The nuclei were counterstained with Hoechst 33342 (Invitrogen), the cells smeared onto microscope slides, air dried and mounted with coverslips using Fluoroshield (Sigma-Aldrich, St. Louis, MO, USA). The immunofluorescent preparations were inspected on an inverted fluorescence microscope (Olympus IX81) and images were captured using Olympus U-CAMD3 camera.

### Virus purification

Purification of PRV was performed according to a protocol developed for purification of MRV, with minor modifications [32, 33]. The samples were kept on ice throughout the protocol. Blood cell pellets and plasma samples with putative high viral load, based on RT-qPCR, flow cytometry and duration of infection, were selected. Blood pellets of 0.55 mL to 1.2 mL were resuspended in Leibovitz’s L15 Medium (Gibco) to a final volume of 5 mL, sonicated five times at 20 kHz for 30 sec with 1 min rest in between. In addition, plasma samples were pooled to generate 5mL plasma samples. To the 5 mL samples, added 100 μL of 10% (w/v) sodium desoxycholate (DOC), vortexed, rested for 5 min, vortexed again and rested for 25 min. Added 2 mL Vertrel XF (DuPont, Wilmington, DE, USA) to the sample, vortexed and sonicated for 30 sec at 20 kHz. Added another 2mL of Vertrel XF, vortexed and sonicated as above, then centrifuged at 9000 g for 10 min at 4°C to separate phases. The top aqueous phase was collected and added 4.5 mL Vertrel XF, vortexed, sonicated and centrifuged as described above. The top aqueous phase was collected and layered onto a 1.22–1.45 g/mL cesium chloride gradient and centrifuged for >12 h at 30 000 rpm using a SW 40TI rotor (Beckman Coulter, Brea, CA, USA) in an Optima LE 80K Ultracentrifuge (Beckman). The tube was punctured to collect the virus band and the density determined by cross referencing the refractive index figure with those computed by Bruner and Vinograd, 1965 [34]. The fraction containing virus band was injected into a Slide-A-Lyzer Dialysis Cassette (Thermo Fisher Scientific) and dialyzed against Dulbecco’s PBS (Sigma-Aldrich,) for >1 h, >3 h and then > 12 h. Dialyzed samples were either stored at -4°C or added glycerol to 15% and stored at -80°C.

#### Transmission electron microscopy and immunogold labelling

Purifed PRV samples were inspected by transmission electron microscopy. Negative staining was performed by adsorbtion of the virus particles to 100 mesh carbon coated Formvar copper grids for 1 min, washed and stained with 4% aqueous uranyl acetate for 1–3 sec. Immunogold labelling was performed at room temperature using the following rabbit sera; Anti-σ1 #K275 [9] and Anti-σ3 #K267 [6]. Briefly, the samples were adsorbed to 100 mesh carbon coated Formvar copper grids for 1 min, incubated with primary antibody for 20 min (diluted 1/50 in 1% fish skin gelatine in PBS) followed by 20 min of proteinA gold (10 nm). Subsequently the grids were stained with 4% aqueous uranyl-acetate. Samples were examined either using a JEM 1400 Electron Microscope (JEOL Ltd, Tokyo, Japan) equipped with a TVIPS TemCam-F216 camera (TVIPS GmbH, Gauting, Germany) or a JEOL 1400Plus Electron Microscope (JEOL Ltd, Tokyo, Japan) equipped with a Ruby camera.

#### Production of polyclonal rabbit antisera against PRV particles

PRV was purified from blood cell pellets and dialyzed as described above and used for production of antisera. High loads of PRV was confirmed by transmission electron microscopy and RT-qPCR. For the first injection, a 0.5 mL batch of purified PRV was mixed with equal amount of Freund’s complete adjuvant and injected subcutaneously on the back of one rabbit. Thereafter, five more injections were administered weekly or biweekly using Freund’s incomplete adjuvant. The rabbit was bled one week after the final injection and the antisera generated was named Anti-PRV (#LUCT).

#### Western blotting

PRV proteins from purified PRV were separated by sodium dodecyl sulfate-polyacrylamide gel electrophoresis (SDS-PAGE), using 12% Bis-Tris Criterion XT gel (Bio-Rad, Hercules, CA, USA). Following SDS-PAGE, the proteins were transferred to a polyvinylidene fluoride (PVDF) membrane (Bio-Rad) and incubated with primary antibody overnight at 4°C using the following sera diluted 1:500; Anti-σ1 #K275 [9], Anti-σ3 #K267 [6], Anti-μ1C #K265 [9], Anti-λ1 (H. Haatveit, Ø. Wessel, T. Markussen et al, submitted for publication) and Anti-PRV #LUCT. After incubation with secondary antibody, anti-rabbit IgG-HRP (Amersham, GE Healthcare, Buchinghamshire,UK) (1:50,000), the PRV proteins were detected by chemiluminescence (ECLPrime Western Blotting Detection Reagent, Amersham). MagicMark (XP Western Protein Standard,Invitrogen) was used as ladder.

#### PRV genome electrophoresis

Total RNA from purified virus originating from both plasma and blood cells was extracted using the combined Trizol LS and RNeasy protocol as described above. RNA was eluted in 50 μL RNase-free water. A sample of the total RNA (20 μL) was ran on 1% agarose gel with cyber safe for 1.5 h at 100 V to separate the genomic segments of PRV.

#### Illumina sequencing, genome assembly and phylogenetic analysis

From the PRV-High inoculum of Challenge experiment #2, total RNA from purified virus was extracted using the combined Trizol LS and RNeasy protocol as described above. A 0.1 volume of 3 M sodium acetate (pH 7.5) and 2X volume of 100% ethanol was added to the eluate (25 μl), and mixed gently. Macrogen (Seoul, Korea) performed library preparation using the TruSeq RNA Library Prep Kit v2 (Illumina Inc., San Diego, CA, USA) and whole genome de novo sequencing (paired-end) on an Illumina HiSeq2000 system.

Paired-end reads were assembled using the PRV genome as a reference [5] and the software BWA v0.6.2 [35]. Deviations from the reference sequence were scored using the software VarScan v2.3.9 [36]. Multiple sequence alignments were performed using AlignX (Vector NTI AdvanceTM 11 package, InfoMax, Inc) and MEGA6 software [37]. Pairwise nucleotide and amino acid sequence identities were calculated using the Sequences Identities and Similarities (SIAS) server (http://imed.med.ucm.es/Tools/sias.html). Both pairwise sequence identities and the phylogenetic tree were constructed using concatenated coding sequences from the present study (NOR2012-V3621) and all PRV genomes available in GenBank (02.12.2016). These sequences originated from the following countries and species: Norway Atlantic salmon (050607, GP-2010, NOR2012-V3621); Canada Atlantic salmon (VT06062012-358, VT06202012-371); Canada Coho salmon (BCJ19943-13); USA Coho salmon (WSKFH12-14); Chile Atlantic salmon (CGA280-05); Japan Coho salmon (PRV-2). The concatenated amino acid sequence sets were constructed using the major gene product from each gene segment only. Except for only minor gaps in the PRV2 Japan L3 and M2, and BCJ19943-13 L1 (the corresponding nucleotides removed from the other strains prior to alignment) complete coding sequences were used in generating the concatenated sequence sets. Maximum likelihood (ML) was used with the T92+G model of nucleotide substitution (best-fit substitution model suggested by the program) [38]. Bootstrap values were calculated from 1000 replicates.

### qPCR; innate immune response

For analysis of innate immune response gene expression in challenge experiment #2, cDNA was prepared from 1000 ng blood RNA using the QuantiTect reverse transcription kit with gDNA elimination (Qiagen) according to the manufacturer’s instructions. Quantitative PCR was performed in triplets on 384-well plates using cDNA corresponding to 5 ng RNA per parallel, SsoAdvanced™ Universal SYBR® Green Supermix, and 500 nM forward and reverse primers targeting type I interferon (IFNab) and the myxovirus resistance GTPase (Mx) as listed in Table 1. The assays were run 40 cycles of 94°C/15 sec, 60°C/30 sec. The specificity of the SYBR green assays was confirmed by melting point analysis. Levels of Elongation factor (EF1α) mRNA were used for normalization by the ΔΔCt method.

### Histopathology

Formalin fixed and paraffin embedded sections of heart and skeletal muscle tissue from challenge experiment #2 were stained with hematoxylin-eosin (HE). Slides were blinded for histopathological examination (n = 144) and evaluated by one investigator. The slides included samples from injected fish collected 2–8 wpc (n = 72) and cohabitant fish collected 4–8 wpc (n = 54) including PRV-High, PRV-Low and positive control group, in addition to the negative control group collected 0–8 wpc (n = 18). The scoring was determined by a visual analog scale (0 to 3) based on the criteria listed in Table 2. In the heart, each compartment was scored separately (epicardium, compactum, spongiosum and atrium), the arithmetic mean was calculated for a total cardiac score. In the skeletal muscle, the red and white portion of the muscle was scored separately.

**Table 2:** Scoring description for histopathological examination of sections.

Score	Pathological description
0	No pathology observed
0 ≥ 1	Mild pathological changes
	Limited number (countable) of inflammatory cells
	<25% of compartment affected)
1 ≥ 2	Moderate pathological changes
	Unlimited number (uncountable) of inflammatory cells
	25–50% of compartment affected
2 ≥ 3	Severe pathological change
	Unlimited number (uncountable) of inflammatory cells
	>50% of compartment affected

#### Immunohistochemistry

A polyclonal antibody against the PRV σ1-protein was used for immunohistochemistry of heart samples using a previously published protocol with minor modifications [9]. Stained slides included injected fish in the PRV-High group of Challenge experiment #2, collected from 2 to 8 wpc (n = 24) and the negative-control group collected at 0 wpc (n = 6)

### Statistics

The Ct-values for PRV in blood and tissues, MFI from flow cytometry and histopathological scoring were compared between injected fish in the PRV-High and PRV-Low group at each time point in challenge experiment #2 using the non-parametric Mann–Whitney test due to the small sample size (n = 6). P-values of p ≤ 0.05 were considered as significant. All statistical analysis described were performed with GraphPad Prism (GraphPad Software Inc., La Jolla, CA, USA).

### Accession number(s)

The complete genome sequence of the PRV strain from the present study (NOR2012-V3621) has been deposited in GenBank under accession numbers KY429943-KY429952.

## Supporting information

S1 Table Pairwise sequence identities. Pairwise nucleotide (above diagonal) and amino acid (below diagonal) sequence identities (%) between concatenated coding regions of PRV. The concatenated amino acid sequence sets were constructed using the major gene product from each gene segment.
(TIF)

S1 Fig One freeze-thaw cycle do not reduce the infectivity of PRV. Viral load in blood cells and plasma of injected fish in the PRV-Low (green) and PRV-Low-Frozen (blue) group (n = 6) in challenge experiment #2. (A) PRV RNA measured by RT-qPCR in blood and plasma, shown as individual and mean Ct-values at each week post challenge (wpc) (B) Amount of PRV σ1- and μNS-protein in blood cells measured by flow cytometry, shown as mean fluorescence intensity (MFI) for individual fish and group mean. Control fish shown in grey. Statistical analysis comparing PRV-High and PRV-Low was performed using Mann-Whitney test at each time point, *p < 0.05, asterisk color (green and blue) indicate the significantly higher group.
(TIF)

S2 Fig Histopathology in the heart of injected fish in challenge experiment #2. Histopathological score of (A) Epicardium, (B) Compactum, (C) Spongiosum and (D) Atrium. Shown as individual score and group mean from 2 to 8 weeks post challenge (wpc) for the PRV-High (red), PRV-Low (green), positive control (black) and negative control (grey) group (n = 6). Statistical analysis comparing PRV-High and PRV-Low was performed using Mann-Whitney test at each time point, *p < 0.05, asterisk color (red and green) indicate the significantly higher group.
(TIF)

## References

[1] Walker PJ, Winton JR. Emerging viral diseases of fish and shrimp. Vet Res. 2010;41(6):51 doi: 10.1051/vetres/2010022 20409453 10.1051/vetres/2010022 PMC2878170

[2] Kongtorp RT, Taksdal T, Lyngøy A. Pathology of heart and skeletal muscle inflammation (HSMI) in farmed Atlantic salmon *Salmo salar*. Dis Aquat Organ. 2004;59(3):217–24. doi: 10.3354/dao059217 15264718 10.3354/dao059217

[3] Kongtorp RT, Halse M, Taksdal T, Falk K. Longitudinal study of a natural outbreak of heart and skeletal muscle inflammation in Atlantic salmon, *Salmo salar* L. J Fish Dis. 2006;29(4):233–44. doi: 10.1111/j.1365-2761.2006.00710.x 16635063 10.1111/j.1365-2761.2006.00710.x

[4] Kongtorp RT, Kjerstad A, Taksdal T, Guttvik A, Falk K. Heart and skeletal muscle inflammation in Atlantic salmon, *Salmo salar* L.: a new infectious disease. J Fish Dis. 2004;27(6):351–8. doi: 10.1111/j.1365-2761.2004.00549.x 10.1111/j.1365-2761.2004.00549.x 15189375

[5] Palacios G, Løvoll M, Tengs T, Hornig M, Hutchison S, Hui J, et al Heart and skeletal muscle inflammation of farmed salmon is associated with infection with a novel reovirus. PLoS One. 2010;5(7):e11487 doi: 10.1371/journal.pone.0011487 20634888 10.1371/journal.pone.0011487 PMC2901333

[6] Markussen T, Dahle MK, Tengs T, Løvoll M, Finstad ØW, Wiik-Nielsen CR, et al Sequence analysis of the genome of piscine orthoreovirus (PRV) associated with heart and skeletal muscle inflammation (HSMI) in Atlantic salmon (*Salmo salar*). PLoS One. 2013;8(7):e70075 doi: 10.1371/journal.pone.0070075 23922911 10.1371/journal.pone.0070075 PMC3726481

[7] Key T, Read J, Nibert ML, Duncan R. Piscine reovirus encodes a cytotoxic, non-fusogenic, integral membrane protein and previously unrecognized virion outer-capsid proteins. J Gen Virol. 2013;94(Pt 5):1039–50. doi: 10.1099/vir.0.048637-0 23343626 10.1099/vir.0.048637-0

[8] Løvoll M, Alarcon M, Bang JB, Taksdal T, Kristoffersen AB, Tengs T. Quantification of piscine reovirus (PRV) at different stages of Atlantic salmon *Salmo salar* production. Dis Aquat Organ. 2012;99(1):7–12. doi: 10.3354/dao02451 22585298 10.3354/dao02451

[9] Finstad ØW, Falk K, Løvoll M, Evensen Ø, Rimstad E. Immunohistochemical detection of piscine reovirus (PRV) in hearts of Atlantic salmon coincide with the course of heart and skeletal muscle inflammation (HSMI). Vet Res. 2012;43(1):27 doi: https://doi.org/10.1186/1297-9716-43-27 22486941 10.1186/1297-9716-43-27 PMC3384478

[10] Finstad OW, Dahle MK, Lindholm TH, Nyman IB, Lovoll M, Wallace C, et al Piscine orthoreovirus (PRV) infects Atlantic salmon erythrocytes. Vet Res. 2014;45(1):35 doi: https://doi.org/10.1186/1297-9716-45-35 24694042 10.1186/1297-9716-45-35 PMC4234517

[11] Wessel Ø, Olsen CM, Rimstad E, Dahle MK. Piscine orthoreovirus (PRV) replicates in Atlantic salmon (Salmo salar L.) erythrocytes ex vivo. Vet Res. 2015;46(26). doi: https://doi.org/10.1186/s13567-015-0154-7 10.1186/s13567-015-0154-7 PMC4350956 25888832

[12] Dahle MK, Wessel O, Timmerhaus G, Nyman IB, Jorgensen SM, Rimstad E, et al Transcriptome analyses of Atlantic salmon (Salmo salar L.) erythrocytes infected with piscine orthoreovirus (PRV). Fish Shellfish Immunol. 2015;45(2):780–90. doi: 10.1016/j.fsi.2015.05.049 26057463 10.1016/j.fsi.2015.05.049

[13] Godoy MG, Kibenge MJ, Wang Y, Suarez R, Leiva C, Vallejos F, et al First description of clinical presentation of piscine orthoreovirus (PRV) infections in salmonid aquaculture in Chile and identification of a second genotype (Genotype II) of PRV. Virol J. 2016;13(98). doi: https://doi.org/10.1186/s12985-016-0554-y 10.1186/s12985-016-0554-y PMC4906990 27296722

[14] Ferguson HW, Kongtorp RT, Taksdal T, Graham D, Falk K. An outbreak of disease resembling heart and skeletal muscle inflammation in Scottish farmed salmon, *Salmo salar* L., with observations on myocardial regeneration. J Fish Dis. 2005;28(2):119–23. doi: 10.1111/j.1365-2761.2004.00602.x 15705157 10.1111/j.1365-2761.2004.00602.x

[15] Di Cicco E, Ferguson HW, Schulze AD, Kaukinen KH, Li S, Vanderstichel R, et al Heart and skeletal muscle inflammation (HSMI) disease diagnosed on a British Columbia salmon farm through a longitudinal farm study. PLoS One. 2017;12(2):e0171471 doi: 10.1371/journal.pone.0171471 28225783 10.1371/journal.pone.0171471 PMC5321275

[16] Marty GD, Morrison DB, Bidulka J, Joseph T, Siah A. Piscine reovirus in wild and farmed salmonids in British Columbia, Canada: 1974–2013. J Fish Dis. 2014;38(8):713–28. doi: 10.1111/jfd.12285 25048977 10.1111/jfd.12285

[17] Garseth AH, Fritsvold C, Opheim M, Skjerve E, Biering E. Piscine reovirus (PRV) in wild Atlantic salmon, *Salmo salar* L., and sea-trout, *Salmo trutta* L., in Norway. J Fish Dis. 2013;36(5):483–93. doi: 10.1111/j.1365-2761.2012.01450.x 23167652 10.1111/j.1365-2761.2012.01450.x

[18] Garver KA, Johnson SC, Polinski MP, Bradshaw JC, Marty GD, Snyman HN, et al Piscine orthoreovirus from western North America is transmissible to Atlantic salmon and Sockeye salmon but fails to cause heart and skeletal muscle inflammation. PLoS One. 2016;11(1):e0146229 doi: 10.1371/journal.pone.0146229 26730591 10.1371/journal.pone.0146229 PMC4701501

[19] Takano T, Nawata A, Sakai T, Matsuyama T, Ito T, Kurita J, et al Full-genome sequencing and confirmation of the causative agent of erythrocytic inclusion body syndrome in coho salmon identifies a new type of piscine orthoreovirus. PLoS One. 2016;11(10):e0165424 doi: 10.1371/journal.pone.0165424 27788206 10.1371/journal.pone.0165424 PMC5082797

[20] Olsen AB, Hjortaas M, Tengs T, Hellberg H, Johansen R. First description of a new isease in rainbow trout (*Oncorhynchus mykiss* (Walbaum)) similar to heart and skeletal muscle inflammation (HSMI) and detection of a gene sequence related to Piscine orthoreovirus (PRV). PLoS One. 2015;10(7):e0131638 doi: 10.1371/journal.pone.0131638 26176955 10.1371/journal.pone.0131638 PMC4503464

[21] Johansen LH, Thim HL, Jorgensen SM, Afanasyev S, Strandskog G, Taksdal T, et al Comparison of transcriptomic responses to pancreas disease (PD) and heart and skeletal muscle inflammation (HSMI) in heart of Atlantic salmon (Salmo salar L). Fish Shellfish Immunol. 2015;46(2):612–23. doi: 10.1016/j.fsi.2015.07.023 26232631 10.1016/j.fsi.2015.07.023

[22] Fredricks DN, Relman DA. Sequence-based identification of microbial pathogens: a reconsideration of Koch's postulates. Clin Microbiol Rev. 1996;9(1):18–33. 8665474 10.1128/cmr.9.1.18 PMC172879

[23] Haatveit HM, Wessel O, Markussen T, Lund M, Thiede B, Nyman IB, et al Viral protein kinetics of piscine orthoreovirus infection in Atlantic salmon blood cells. Viruses. 2017;9(3). doi: https://org/10.3390/v9030049 10.3390/v9030049 PMC5371804 28335455

[24] Collet B. Innate immune responses of salmonid fish to viral infections. Dev Comp Immunol. 2014;43(2):160–73. doi: 10.1016/j.dci.2013.08.017 23981327 10.1016/j.dci.2013.08.017

[25] Attoui H, Fang Q, Mohd JF, Cantaloube JF, Biagini P, de Micco P, et al Common evolutionary origin of aquareoviruses and orthoreoviruses revealed by genome characterization of Golden shiner reovirus, Grass carp reovirus, Striped bass reovirus and golden ide reovirus (genus *Aquareovirus*, family *Reoviridae*). J Gen Virol. 2002;83(Pt 8):1941–51. doi: 10.1099/0022-1317-83-8-1941 12124458 10.1099/0022-1317-83-8-1941

[26] Nibert ML, Duncan R. Bioinformatics of recent aqua- and orthoreovirus isolates from fish: Evolutionary gain or loss of FAST and fiber proteins and taxonomic implications PLoS One. 2013;8(7):e68607 doi: 10.1371/journal.pone.0068607 23861926 10.1371/journal.pone.0068607 PMC3701659

[27] Attoui H, Mertens PPC, Becnel J, Belaganahalli S, Bergoin M, Brussaard CP, et al Reoviridae In: King AMQ, Adams MJ, Carstens EB, Lefkowitz EJ, editors. Virus taxonomy: Ninth report of the international committee on taxonomy of viruses. Amsterdam: Academic Press; 2012 p. 546–60.

[28] Kibenge M, Iwamoto T, Wang Y, Morton A, Godoy M, Kibenge F. Whole-genome analysis of piscine reovirus (PRV) shows PRV represents a new genus in family Reoviridae and its genome segment S1 sequences group it into two separate sub-genotypes. Virol J. 2013;10(1):230 doi: https://org/10.1186/1743-422X-10-230 23844948 10.1186/1743-422X-10-230 PMC3711887

[29] Siah A, Morrison DB, Fringuelli E, Savage P, Richmond Z, Johns R, et al Piscine reovirus: Genomic and molecular phylogenetic analysis from farmed and wild salmonids collected on the Canada/US Pacific coast. PLoS One. 2015;10(11):e0141475 Epub 2015/11/05. doi: 10.1371/journal.pone.0141475 26536673 10.1371/journal.pone.0141475 PMC4633109

[30] Garseth ÅH, Ekrem T, Biering E. Phylogenetic evidence of long distance dispersal and transmission of piscine reovirus (PRV) between farmed and wild Atlantic salmon. PLoS One. 2013;8(12). doi: https://org/10.1371/journal.pone.0082202 10.1371/journal.pone.0082202 PMC3859594 24349221

[31] Lund M, Rosaeg MV, Krasnov A, Timmerhaus G, Nyman IB, Aspehaug V, et al Experimental Piscine orthoreovirus infection mediates protection against pancreas disease in Atlantic salmon (Salmo salar). Vet Res. 2016;47(1):107 doi: 10.1186/s13567-016-0389-y 27769313 10.1186/s13567-016-0389-y PMC5075195

[32] Mendez II, Hermann LL, Hazelton PR, Coombs KM. A comparative analysis of freon substitutes in the purification of reovirus and calicivirus. J Virol Methods. 2000;90(1):59–67. doi: https://doi.org/10.1016/S0166-0934(00)00217-2 11011081 10.1016/s0166-0934(00)00217-2

[33] Berard A, Coombs KM. Mammalian reoviruses: propagation, quantification, and storage. Current protocols in microbiology. 2009;Chapter 15:Unit15C.1. doi: https://doi.org/10.1002/9780471729259.mc15c01s14 10.1002/9780471729259.mc15c01s14 19653214

[34] Bruner R, Vinograd J. The evaluation of standard sedimentation coefficients of sodium RNA and sodium DNA from sedimentation velocity data in concentrated NaCl and CsCl solutions. Biochim Biophys Acta. 1965;108(1):18–29. 5862239 10.1016/0005-2787(65)90104-8

[35] Li H, Durbin R. Fast and accurate short read alignment with Burrows-Wheeler transform. Bioinformatics. 2009;25(14):1754–60. doi: 10.1093/bioinformatics/btp324 19451168 10.1093/bioinformatics/btp324 PMC2705234

[36] Koboldt DC, Zhang Q, Larson DE, Shen D, McLellan MD, Lin L, et al VarScan 2: somatic mutation and copy number alteration discovery in cancer by exome sequencing. Genome Res. 2012;22(3):568–76. doi: 10.1101/gr.129684.111 22300766 10.1101/gr.129684.111 PMC3290792

[37] Tamura K, Stecher G, Peterson D, Filipski A, Kumar S. MEGA6: Molecular Evolutionary Genetics Analysis version 6.0. Mol Biol Evol. 2013;30(12):2725–9. doi: 10.1093/molbev/mst197 24132122 10.1093/molbev/mst197 PMC3840312

[38] Tamura K. Estimation of the number of nucleotide substitutions when there are strong transition-transversion and G+C-content biases. Mol Biol Evol. 1992;9(4):678–87. 1630306 10.1093/oxfordjournals.molbev.a040752

