# Supplementary figures and images for: Infection with purified Piscine orthoreovirus demonstrates a causal relationship with heart and skeletal muscle inflammation in Atlantic salmon
Source: PLoS One. 2017 Aug 25;12(8):e0183781. doi: 10.1371/journal.pone.0183781 (PMC5571969; doi:10.1371/journal.pone.0183781)

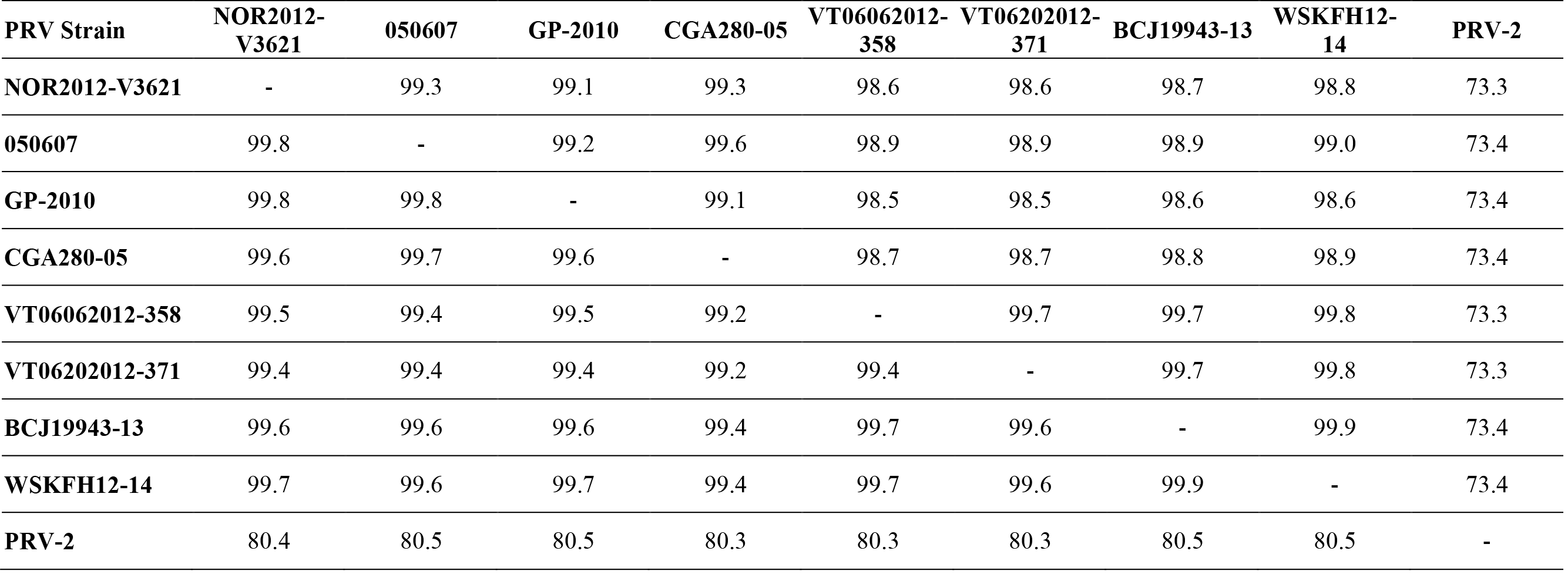

Supplement: S1 Table — Pairwise nucleotide (above diagonal) and amino acid (below diagonal) sequence identities (%) between concatenated coding regions of PRV. The concatenated amino acid sequence sets were constructed using the major gene product from each gene segment. (TIF) [file pone.0183781.s001.tif]

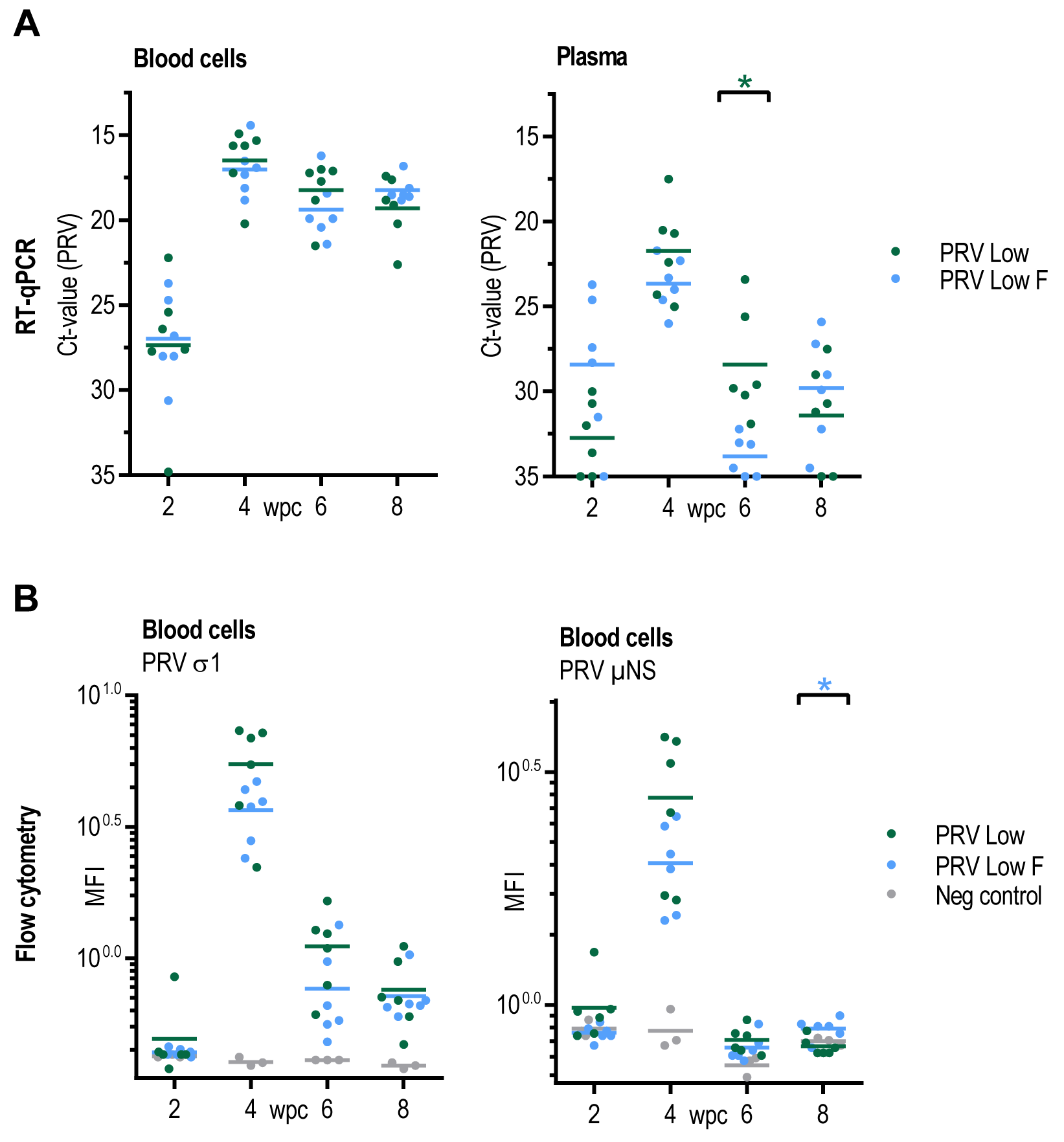

Supplement: S1 Fig — Viral load in blood cells and plasma of injected fish in the PRV-Low (green) and PRV-Low-Frozen (blue) group (n = 6) in challenge experiment #2. (A) PRV RNA measured by RT-qPCR in blood and plasma, shown as individual and mean Ct-values at each week post challenge (wpc) (B) Amount of PRV σ1- and μNS-protein in blood cells measured by flow cytometry, shown as mean fluorescence intensity (MFI) for individual fish and group mean. Control fish shown in grey. Statistical analysis comparing PRV-High and PRV-Low was performed using Mann-Whitney test at each time point, *p < 0.05, asterisk color (green and blue) indicate the significantly higher group. (TIF) [file pone.0183781.s002.tif]

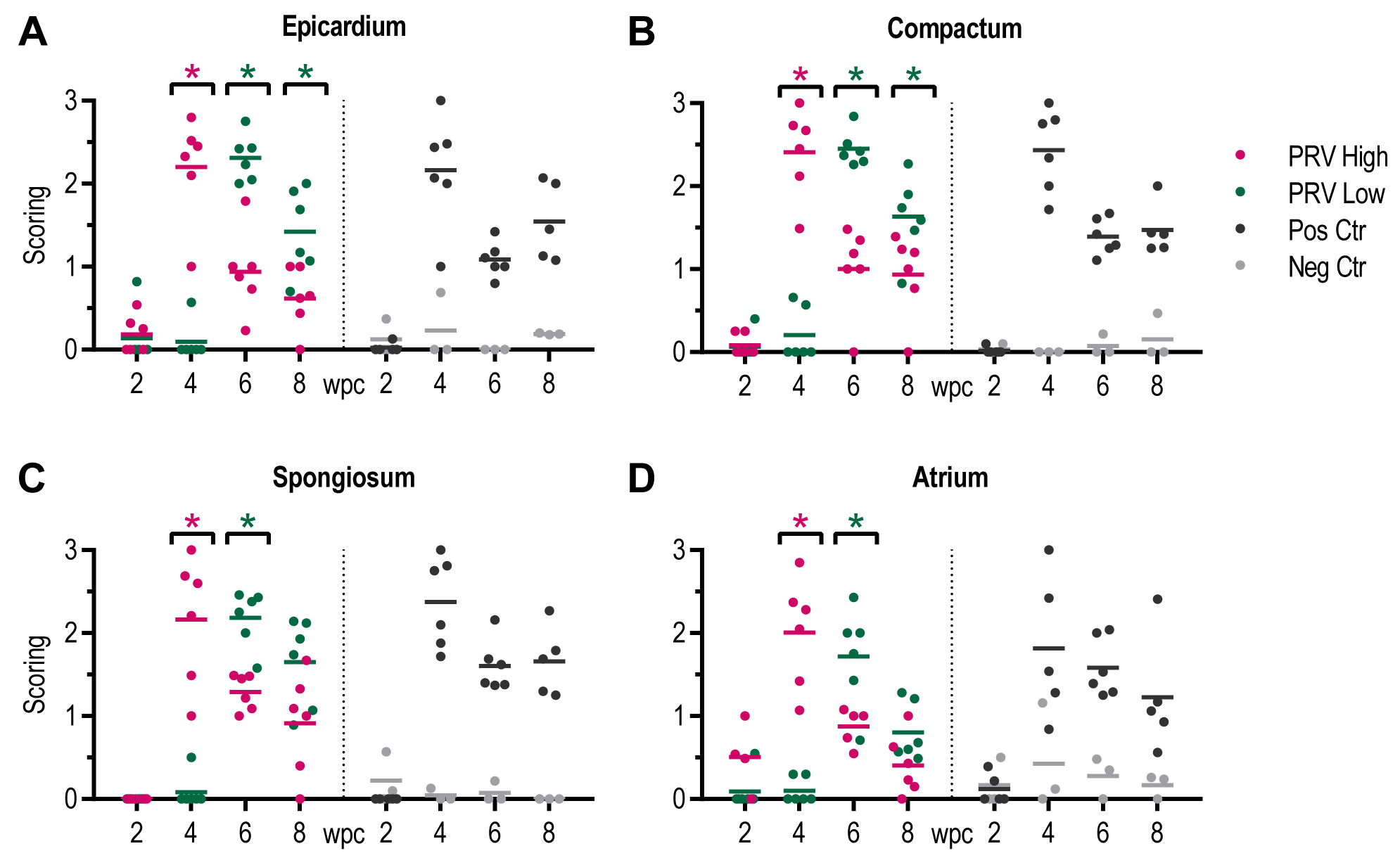

Supplement: S2 Fig — Histopathological score of (A) Epicardium, (B) Compactum, (C) Spongiosum and (D) Atrium. Shown as individual score and group mean from 2 to 8 weeks post challenge (wpc) for the PRV-High (red), PRV-Low (green), positive control (black) and negative control (grey) group (n = 6). Statistical analysis comparing PRV-High and PRV-Low was performed using Mann-Whitney test at each time point, *p < 0.05, asterisk color (red and green) indicate the significantly higher group. (TIF) [file pone.0183781.s003.tif]
